# In Situ Growth of Hierarchical Silver Sub‐Nanosheets on Zinc Nanosheets‐Based Hollow Fiber Gas‐Diffusion Electrodes for Electrochemical CO_2_ Reduction to CO

**DOI:** 10.1002/smsc.202400184

**Published:** 2024-07-10

**Authors:** Guoliang Chen, Lei Ge, Yizhu Kuang, Hesamoddin Rabiee, Beibei Ma, Fatereh Dorosti, Ashok Kumar Nanjundan, Zhonghua Zhu, Hao Wang

**Affiliations:** ^1^ Centre for Future Materials University of Southern Queensland Springfield QLD 4300 Australia; ^2^ School of Engineering University of Southern Queensland Springfield QLD 4300 Australia; ^3^ School of Chemical Engineering The University of Queensland Brisbane QLD 4072 Australia

**Keywords:** charge transfer, electrochemical reduction of CO_2_, hierarchically silver sub‐nanosheets, hollow fiber gas diffusion electrode

## Abstract

Electrochemical reduction of CO_2_ (CO_2_RR) is an effective strategy to mitigate carbon emission effects and store renewable electricity in value‐added feedstocks, but it still suffers low production rate and current density. A nanostructured catalyst offers opportunities to enhance CO_2_RR activity by contributing numerous active sites and promoting charge transfer. Herein, a Cu hollow fiber gas diffusion electrode (HFGDE) with silver sub‐nanosheets on a zinc nanosheet structure to produce CO is reported. Compared to the HFGDE only possessed zinc nanosheet structure, the as‐prepared HFGDE with hierarchical sub‐nano AgZn bimetal nanosheets exhibits a twice‐partial current density of CO and a CO production rate at the applied potential −1.3 V (versus reversible hydrogen electrode). The unique Ag sub‐nanosheets interconnected Zn nanosheets provide multiple charge transfer channels, and the synergistic effect between Ag and Zn improves the adsorption binding energy of COOH* intermediate, resulting in a lower charge transfer resistance and fast CO_2_RR kinetics to produce CO. This research demonstrates the high potential of nanoengineering electrocatalysts for HFGDE to achieve highly efficient CO_2_ reduction.

## Graphical abstract

A hierarchically nanostructured AgZn bimetal catalyst with reduced charge transfer resistance, incorporating a hollow fiber electrode with excellent gas diffusion properties, is an outstanding strategy to improve CO_2_RR performance in aqueous electrolytes. The Ag30ZnNS‐HF electrode achieves a high CO partial current density of 82.5 mA cm^−2^ and a CO production rate of 1364.5 μmol h^−1^ cm^−2^.

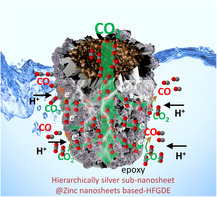

## 1 Introduction

Utilizing renewable electricity to drive the electrochemical reduction reaction of CO_2_ (CO_2_RR) into carbon‐based fuels and value‐added chemical stocks is an effective strategy for mitigating the carbon emission effect and reducing feedstock consumption.^[^ ^1^ ^]^ Among various value‐added products derived from CO_2_RR, CO draws intensive attention as an essential chemical feedstock for industrial production, such as the Fischer–Tropsch process.^[^ ^2^ ^]^ However, due to the more negative reduction potential required for CO_2_RR to produce CO, the hydrogen evolution reaction (HER) also occurs on the cathode. Therefore, a highly efficient electrocatalyst requires high intrinsic activity and selectivity for CO production. Meanwhile, a suitable electrode configuration is also essential to improve the CO yield and current density.

Recently, the application of a hollow fiber gas diffusion electrode (HFGDE) or hollow fiber gas penetration electrode has dramatically improved the kinetics of CO_2_RR and increased current density.^[^ ^3^ ^]^ The CO_2_ gas builds up pressure in the lumen side of the hollow fiber, penetrating through the porous hollow fiber wall and reaching the catalyst/electrolyte interfaces to provide a high CO_2_ concentration near the electrode. This differs from planar electrodes working in H‐Cell, where CO_2_ dissolves in the bulk electrolyte and diffuses to the catalyst/electrolyte interfaces, resulting in a sluggish reaction kinetic and a low current density due to the long CO_2_ diffuse path to the active sites.^[^ ^4^ ^]^ The HFGDE does not require a separate gas chamber and flow cell to achieve gas diffusion, reducing the complexity of assembling a conventional GDE that consists of a superhydrophobic macroporous layer, microporous layer, and catalyst layer. These unique properties of HFGDEs make them a high potential for achieving industrial lever current density of more than 200 mA cm^−2^. The fabrication of HFGDEs can be carried out in a facile dry‐wet extrusion process, and a hundred meters of green fiber can be produced in one batch, showing a high potential for large‐scale manufacturing.^[^ ^5^ ^]^ It is also feasible to scale up HF‐type electrodes by making HF‐lined or circled arrays for industrial applications.^[^ ^6^ ^]^ The HFGDE with small radial dimensions and high surface area‐to‐volume ratios could enable them to load various electrocatalysts and increase the dense pack for large‐scale applications.

To date, precious metals, such as Au and Ag exhibit high CO selectivity and have been intensively studied.^[^ ^7^ ^]^ Additionally, earth‐abundant Zn can be a promising metal to replace the high cost of precious metals for the production of CO.^[^ ^8^ ^]^ Nanostructured Zn such as Zn dendrites,^[^ ^9^ ^]^ Zn nanosheets,^[^ ^10^ ^]^ porous Zn,^[^ ^11^ ^]^ and hexagonal Zn nanoplates^[^ ^12^ ^]^ have been designed to improve the activity and selectivity of Zn catalysts. Compared to bulk metal catalysts with smooth surfaces, nanostructured catalysts could provide numerous active sites, more edge/low coordinated sites, and induced geometric effects, boosting CO_2_RR activity. For example, the n‐Zn electrode with abundant zinc nanosheet structures achieved about three times the CO Faradaic efficiency (FE) than Zn foil.^[^ ^13^ ^]^ However, a considerable ratio of Zn (002) facets still contribute to HER. Therefore, introducing another metal to Zn is a promising method to improve the activity and selectivity of zinc‐based catalysts. The bimetallic method could alter the binding energy for the adsorption of the COOH* intermediate to improve the CO_2_RR kinetics and product selectivity.^[^ ^14^ ^]^ Among the metal candidates, Ag exhibits a high selectivity of CO, and nanostructure Ag catalyst can lower the required overpotential for CO production. An Ag–Zn bimetal catalyst would have an improved CO_2_RR activity than Ag or Zn single metal as the previous study reported that the Ag and Zn possessed a weaker and stronger COOH* binding than Au in the volcano trend plot.^[^ ^15^ ^]^ In a design ZnO–Ag@UC catalyst, the heterointerface between Ag and ZnO sparks the electron transfer from Zn to Ag, increasing bonding strength to the COOH* intermediate.^[^ ^16^ ^]^ In another similar research, the incorporation of Cu in Zn foil via galvanic replacement reaction (GRR) engenders the electron effect that donates electrons from Zn to Cu, and the Zn–Cu catalyst outperformed pure Cu or Zn due to the excellent synergistic catalytic activity of Zn–Cu.^[^ ^17^ ^]^ Overall, the CO_2_RR performance of Zn‐based catalysts still needs to be improved, and designing a ZnAg bimetal catalyst with hollow fiber gas diffusion electrode configuration seems promising for efficient electrochemical conversion of CO_2_ to CO.

Herein, we design a novel HFGDE with in situ growth of hierarchically sub‐nano interconnected AgZn bimetal nanosheets to further boost the electrochemical conversion of CO_2_ to CO. The pulse electrodeposition technique was applied to grow the zinc nanosheets, and the GRR method was applied to the in situ growth of silver sub‐nanosheets. The hierarchical silver sub‐nanosheets interconnected with zinc nanosheets could provide multiple electrotransfer channels to lower the charge transfer resistance and increase the charge density. This unique structure also provided the increased electrochemical active surface area (ECSA), and the synergistic interaction between Ag and Zn resulted in stronger COOH* binding energy adsorption to improve the CO_2_RR kinetics and CO selectivity. The HF electrode with hierarchically interconnected AgZn sub‐nanosheet structure achieved a high CO partial current density of 82.5 mA cm^−2^ and a CO product rate of 1364.6 μmol h^−1^ cm^−2^, more than twice each of the electrodes with only zinc nanosheet structure. Moreover, conducting this electrode in non‐GDE mode resulted in much lower partial current densities of CO, indicating the significance of HFGDE configuration for high activity of CO_2_RR performance through providing high CO_2_ local concentration and maximizing triphasic interfaces.

## 2 Results and Discussion

### 2.1 Microstructure and Morphology Analysis

**Figure 1:**
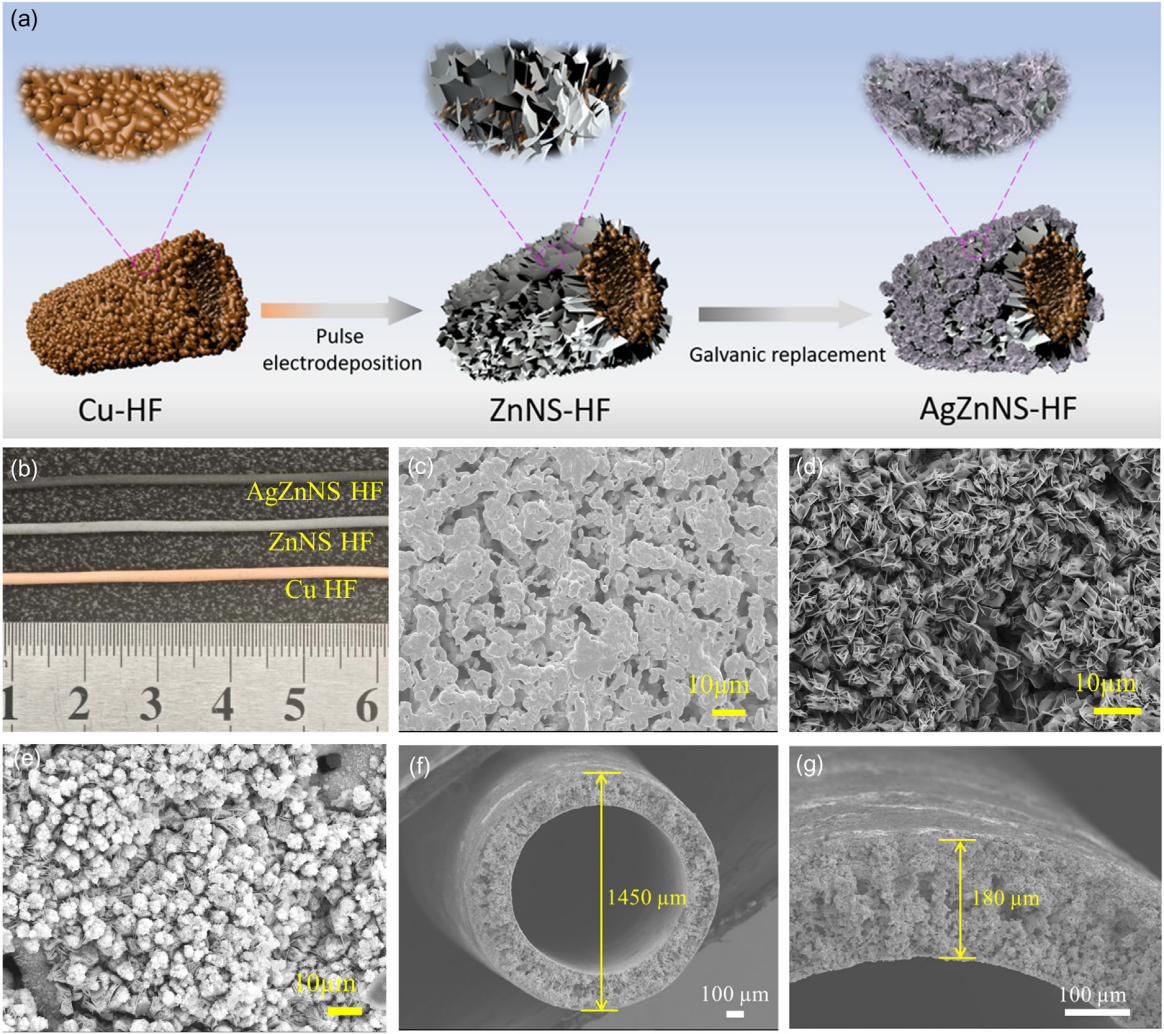
a) Schematic illustration of preparing AgZnNS‐HF; b) optical photo images of Cu HF, ZnNS‐HF, and AgZnNS‐HF; surface SEM images of c) Cu HF, d) ZnNS‐HF, and e) AgZnNS‐HF; f,g) cross‐section SEM images of Cu HF.

The in situ growth of silver sub‐nanosheets on zinc nanosheets‐based Cu HF is via two steps and is illustrated in **Figure** **1**a. The ZnNS‐HF was first prepared via pulse electrodeposition of zinc nanosheets on Cu HF, and the copper color disappeared with the Cu HF turning to silver gray (Figure 1b) after the electrodeposition process, which indicates a uniform zinc nanosheet layer coating on the outer surface of Cu HF. From Figure 1c and d, different surface morphology was observed, with the Cu HF possessing an interconnected microporous network and the ZnNS‐HF covering with a well‐connected zinc nanosheets layer. The applied pulse electrodeposition technique plays a vital role in keeping the concentration of zinc ions near the electrode, thus stabilizing the nucleating and crystal‐growing rate of zinc ions to form uniform sizes of zinc nanosheets.^[^ ^18^ ^]^ This zinc nanosheet structure could provide an enlarged zinc surface area for the GRR of silver. The reduction of Ag ions by Zn nanosheets takes place as follows: Zn + 2Ag^+^ = 2Ag + Zn^2+^ (*E* ^0^_Ag+/Ag_ =+0.799 V > *E* ^0^_Zn2+/Zn_ = −0.726 V). The silver‐gray color of ZnNS‐HF changed to black once the ZnNS‐HF was immersed in the silver ions solution (Figure 1b). Flower‐like silver sub‐nanosheet clusters were formed between the zinc nanosheet network (Figure 1e), and the resulted HF named AgZnNS‐HF. For example, the Ag30ZnNS‐HF is named by placing the ZnNS‐HF in silver ion solution for a GRR time of 30 s. The hierarchically silver sub‐nanosheets supported on a well‐connected zinc nanosheet network could potentially increase the ECSA and promote CO_2_ delivery to the electrolyte/catalyst interface, which could create more triple‐phase interfaces and improve the electrochemical conversion of CO_2_.

Cu HF with abundant microporous structures is a good gas diffuser, and the gas delivery property of the electrode is critical in stabilizing high CO_2_ local concentration. The Cu HF was fabricated by a phase inversion and sintering process (schematically shown in Figure S1, Supporting Information), and the interconnected microporous network was formed by removing the polymer binder. The diameter of Cu HF is around 1.45 mm (Figure 1f) with a uniform wall thickness of 180 μm (Figure 1g). The well‐connected metallic and porous network through the inner surface and outer surface of Cu HF enables it to be a good gas diffuser to promote CO_2_ gas transportation. The gas permeability of Cu HF was around 3.39 ± 0.03 × 10^+6^ Barrer (Equation S3, Supporting information) when a 30 mL min^−1^ CO_2_ flow rate was applied. From cross‐section scanning electron microscopy (SEM) images of Figure S2a and b, Supporting Information, the catalyst layer thickness of ZnNS‐HF and Ag30ZnNS‐HF is around 11 and 12 μm, respectively. To investigate whether the coating of a thick catalyst layer could affect the gas delivery property of hollow fiber electrode, we also calculated the gas permeability of these two samples, 3.23 ± 0.05 × 10^+6^ Barrer for ZnNS‐HF and 3.13 ± 0.05 × 10^+6^ Barrer for Ag30ZnNS‐HF. The gas permeability slightly decreased compared to the value of the original Cu HF, confirming that the catalyst coverage did not lead to the blockage of HF pores and could retain the excellent gas penetrating property of hollow fiber and feed sufficient CO_2_ for the reaction sites.

**Figure 2:**
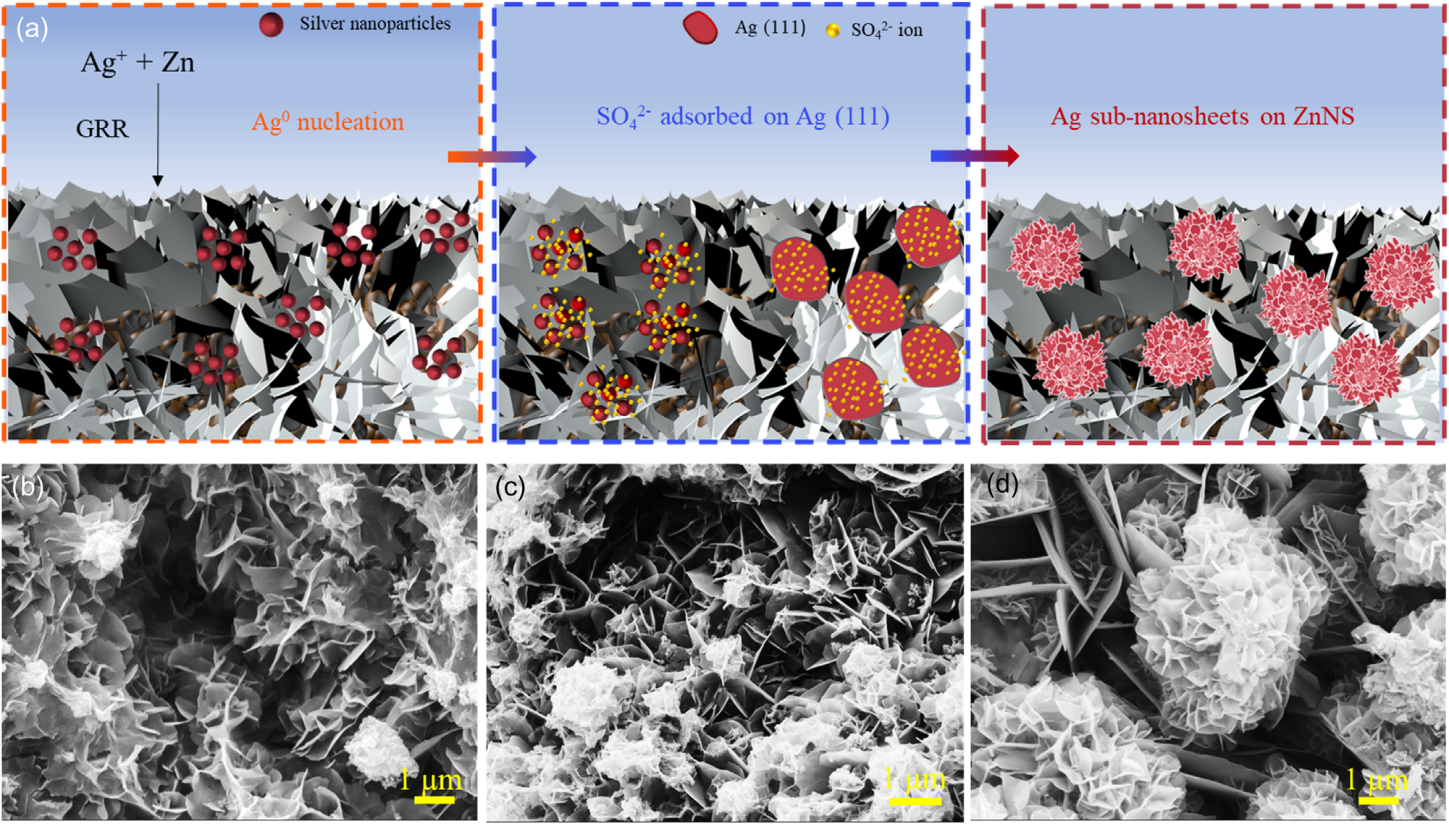
a) Schematic illustration of the formation of silver sub‐nanosheets on zinc nanosheets‐based Cu HF; surface SEM images of b) Ag5ZnNS‐HF, c) Ag10ZnNS‐HF, and d) Ag30ZnNS‐HF.

The in situ growth of silver sub‐nanosheets is illustrated in **Figure** **2**a, and the process mainly includes three stages: the Ag^0^ nucleation, the shaping stage of SO_4_^2−^ ions adsorbed on the Ag (111) plane to force the growth of anisotropically structured nanoplates, and the formation of flower‐like silver sub‐nanosheets. In detail, the GRR happens once the ZnNS‐HF is immersed in the silver ions solutions, and the silver ions will be reduced to silver atoms while the Zn atom turns to the Zn ions diffusing to the solution. When the concentration of Ag^0^ has reached the supersaturation level, they will start to nucleate, forming the thermodynamic stable faceted nanoparticles exposing the Ag (111) facet with the low surface energy.^[^ ^19^ ^]^ The SO_4_^2−^ ions with small particle sizes are likely to adsorb onto the surfaces of Ag (111) and coordinate silver nuclei, thereby inhibiting them from aggregating through electrostatic stabilization.^[^ ^20^ ^]^ Therefore, Ag nuclei grow into small nanoplates due to the preferential adsorption of SO_4_^2−^ ions on the Ag (111) planes. In the shaping process, the SO_4_^2−^ ions play a role similar to those conventional capping agents, such as polyvinylpyrrolidone molecules that bind strongly to the Ag (100) planes,^[^ ^21^ ^]^ critic acid molecules^[^ ^22^ ^]^ and borate ions^[^ ^23^ ^]^ adsorbed on the Ag (111) planes.

To further verify the growth mechanism of the silver sub‐nanosheets, the silver sub‐nanosheets at different stages were characterized by SEM. In the nucleation period, small Ag nanoparticles and Ag particle clusters were observed on the zinc nanosheets layer after immersing the ZnNS‐HF into the mixed solution (10 mM AgNO_3_ + 5 mM K_2_SO_4_) for 5 s (Figure 2b). In the shaping process, the coexistence of silver nanoparticle clusters and sub‐nanosheets can be found in the 10 s of galvanic replacement process (Figure 2c). After the GRR process for 30 s, the silver nanoparticles disappeared, and the sub‐nanosheets with a hierarchical structure emerged (Figure 2d). The energy‐dispersive X‐ray (EDX) spectroscopy elemental image (Figure S3, Supporting Information) of Ag and Zn on the outer surface shows that the silver sub‐nanosheets are dispersed on the zinc nanosheets layer. With the increase of GRR time, the surface atomic ratio of Ag for AgZnNS‐HF samples increased (Figure S4, Supporting Information). The surface atomic ratio reaches 24.4% for the Ag30ZnNS‐HF sample. The ZnNS‐HF (Figure S5a, Supporting Information) showed a dense zinc nanosheet morphology, and small silver particle clusters were observed in the sample with 1 s galvanic replacement time (Figure S5b, Supporting Information). The loose and dendrite‐like morphology started to form when the GRR time reached 60 s (Figure S5c and d, Supporting Information), and a high‐dense dendrite structure formed after the GRR process of 5 min (Figure S5e, Supporting Information). The formation of loose and dendrite‐like silver structures is mainly due to the high concentration of Ag^0^ produced during the long‐time galvanic replacement process, accelerating the nucleation rate of silver ions.^[^ ^24^ ^]^ Along with the loading quantity of silver, the interaction between silver nanosheets and zinc nanosheets also needs to be considered to stand the CO_2_ gas pressure. The loose structure of silver will be flushed out or dispatched after a certain flow rate. The intact silver sub‐nanosheets‐connected zinc nanosheets network was only formed with a maximum GRR time of 30 s, which is also the maximum amount of silver loading.

The SO_4_^2−^ is critical for regulating the morphology of silver nanocrystals via adsorbing on the Ag (111) planes. We also investigated the concentration effects of SO_4_^2−^ ions by immersing the ZnNS‐HF in 10 mM AgNO_3_ + 2.5 mM K_2_SO_4_ solution for the 30 s, and the coexisting of silver particle clusters and silver sub‐nanosheets is observed in Figure S5f, Supporting Information; this means a relatively high concentration of SO_4_^2−^ ions could accelerate the formation of silver sub‐nanosheets. Other than that, different concentrations of silver ions without SO_4_^2−^ addition were also conducted; only silver nanoparticle clusters were observed on the zinc nanosheets layer with a GRR period of 30 s at 1 mM (Figure S5g, Supporting Information), 3 mM (Figure S5h, Supporting Information), and 5 mM (Figure S5i, Supporting Information) AgNO_3_ solution. The existence of SO_4_^2−^ ions is vital for preventing the silver particle aggregation and inhibiting the growth along the Ag (111) plane. Furthermore, to investigate whether the zinc nanosheet structure could affect the growth of silver sub‐nanosheets, we prepared a flat surface zinc hollow fiber by carefully scratching the outer surface of ZnNS‐HF with a sharp blade. The flat surface zinc hollow fiber was immersed in the 10 mM AgNO_3_ + 5 mM K_2_SO_4_ solution for 30 s. From Figure S5j, k, and l, Supporting Information, only a small amount of silver particle clusters was observed on the flat zinc surface. The failure growth of silver sub‐nanosheets is probably attributed to the abundant anisotropic Zn (002) crystal facets on the ZnNS‐HF surface, which could help inhibit silver crystal growth along the Ag (111) direction, and the nanosheet pore voids could provide restricted rooms for flower‐like silver sub‐nanosheets growth.

Lastly, considering the GRR also happens between Cu and Ag^+^, Cu + 2Ag^+^ = 2Ag + Cu^2+^ (*E* ^0^_Ag+/Ag_ = +0.799 V >*E* ^0^_Cu2+/Cu_ = + 0.34 V). The Cu hollow fibers were immersed in 10 mM AgNO_3_ + 5 mM K_2_SO_4_ solution for different periods of 5 s (Figure S6a, Supporting Information), 10 s (Figure S6b, Supporting Information), and 30 s (Figure S6c, Supporting Information), and in 10 mM AgNO_3_ solution for 30 s (Figure S6d, Supporting Information). The leaf‐like silver structure increases with the GRR time from 5 to 30 s, and the dendrite morphology with large Ag particle sizes emerged in the Cu HF immersed in the 10 mM AgNO_3_ solution, indicating higher GRR rates without the assistance of SO_4_^2−^. The failure growth of silver nanosheets on the surface of Cu HF is due to the difference in reaction rates between Cu metal and Ag^+^ (compared to Zn metal and Ag^+^) and the smooth surface of the Cu particles. In a similar study, copper nanosheets were obtained by immersing Zn foil in different concentrations of CuSO_4_ solution for varied periods.^[^ ^25^ ^]^ In conclusion, the SO_4_^2−^ ions are good candidates for stabilizing the Ag (111) and inhibiting the growth along the Ag (111), and different GRR rates (e.g., metal types, the concentration of metal ions), as well as the morphology of substrate, could lead to different silver morphology.

**Figure 3:**
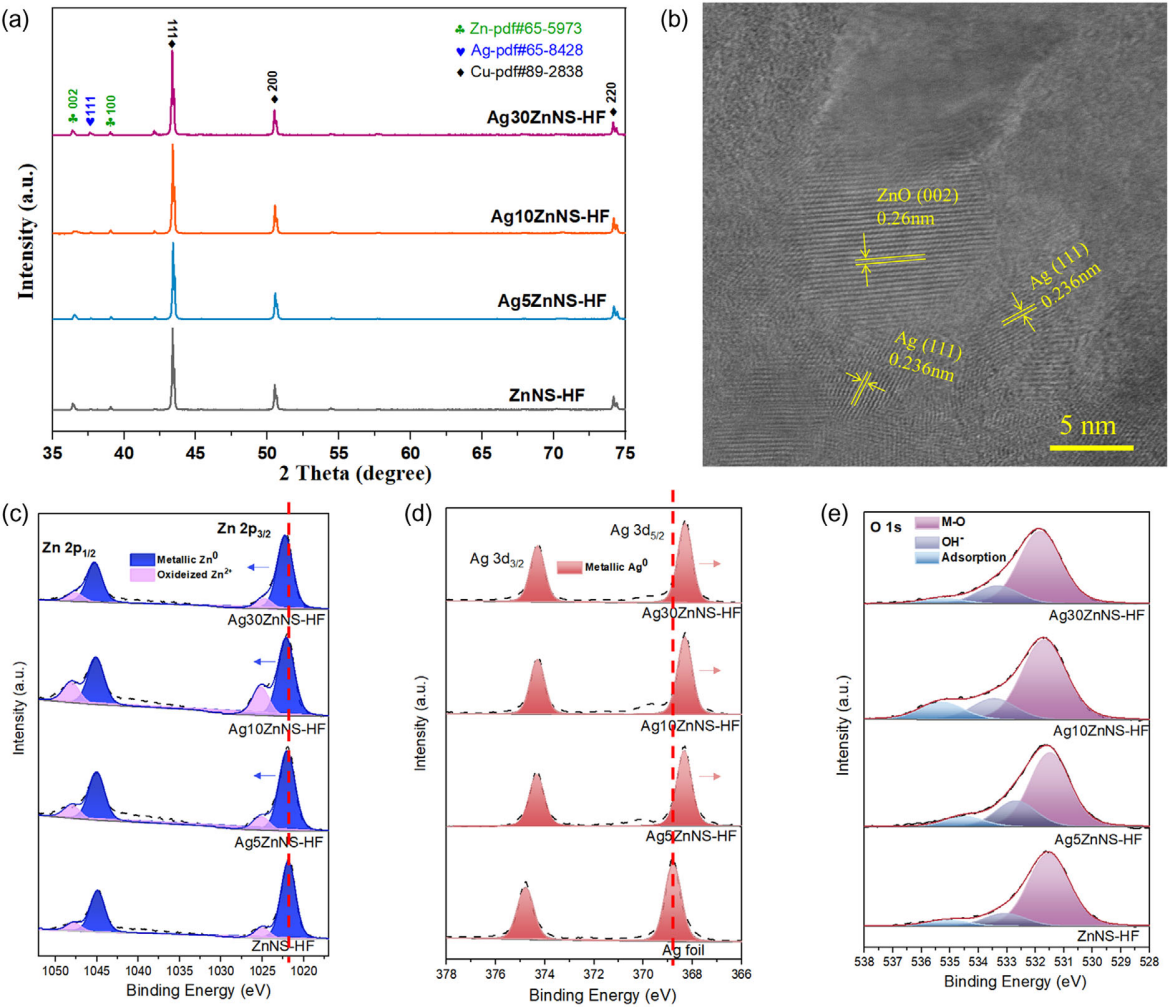
a) XRD patterns of ZnNS‐HF, Ag5ZnNS‐HF, Ag10ZnNS‐HF, and Ag30ZnNS‐HF; b) HR‐STEM image of Ag30ZnNS‐HF; c) Zn 2*p* XPS spectra of ZnNS‐HF, Ag5ZnNS‐HF, Ag10ZnNS‐HF, and Ag30ZnNS‐HF; d) Ag 3*d* XPS spectra of Ag foil, Ag5ZnNS‐HF, Ag10ZnNS‐HF, and Ag30ZnNS‐HF; and e) O 1*s* XPS spectra of ZnNS‐HF, Ag5ZnNS‐HF, Ag10ZnNS‐HF, and Ag30ZnNS‐HF.

X‐Ray diffraction (XRD) patterns of bulk phase ZnNS‐HF, Ag5ZnNS‐HF, Ag10ZnNS‐HF, and Ag30ZnNS‐HF are shown in **Figure** **3**a. Three copper metallic peaks at 43.3°, 50.4°, and 74.1° are detected in all samples, corresponding to Cu (111), (200), and (220) crystal planes of Cu (PDF#89‐2848).^[^ ^26^ ^]^ The results are in accordance with the peaks observed in the origin Cu HF (Figure S7a, Supporting Information), and we can conclude that the copper crystal phase does not change after the electrodeposition of zinc nanosheets on the Cu HF outer surface. The main zinc characteristic diffraction peaks at 36.3° and 39.0° corresponding to (002) and (100) crystal planes of Zn (PDF#65‐5973), which confirm the formation of metallic zinc layer during the electrodeposition process.^[^ ^27^ ^]^ The emergence of the Ag (111) crystal plane (PDF#65‐8428) at a diffraction peak of 37.7° in the Ag5ZnNS‐HF, Ag10ZnNS‐HF, and Ag30ZnNS‐HF shows the successful growth of silver sub‐nanosheets structure on the zinc nanosheets network. The low intensity of the Ag (111) peak is probably attributed to the low concentration of silver on the electrode surface and the relatively high intensity of the copper diffraction peak from the bulk Cu HF.^[^ ^28^ ^]^ The high‐resolution scanning transmission electron microscopy (HR‐STEM) image (Figure 3b) further confirmed the existence of Ag as the characteristic spacing of 0.236 nm is attributed to the Ag (111) in the Ag30ZnNS‐HF sample.

To further investigate the electronic states of silver sub‐nanosheets on zinc nanosheets based on Cu HF, we analyzed with the high‐resolution X‐Ray photoelectron spectroscopy (XPS) with XPS spectra of Cu 2*p* (Figure S6b, S7, Supporting Information), Zn 2*p* (Figure 3c), Ag 3*d* (Figure 3d), and O 1*s* (Figure 3e). The detected Cu 2*p* peak at around 932.5 eV in Cu HF (Figure S7b, Supporting Information) completely disappeared (Figure S8, Supporting Information), indicating a uniform Zn/AgZn catalyst layer covering the Cu HF outer surface. The XPS spectra of Zn 2*p*_3/2_ and Zn 2*p*_1/2_ peaks for the ZnNS‐HF sample can each be deconvoluted into two peaks (Figure 3c), and the relatively low binding energy peak at around 1021.9 and 1044.9 eV is associated with the metallic Zn (Zn^0^ state). In contrast, the relatively high binding energy peaks 1025.0 and 1047.8 eV are linked to the oxidized zinc (Zn^2+^ state). The Zn 2*p*3/2 spectra of the Ag5ZnNS‐HF, the Ag10ZnNS‐HF, and the Ag30ZnMP‐HF samples are shifted slightly toward higher binding energies, 1022.0, 1022.1, and 1022.3 eV, respectively. The shifts of the XPS Zn 2*p*_3/2_ to higher binding energies indicate electron density depletion around the Zn atoms.^[^ ^29^ ^]^ The Ag 3*d* spectrum (Figure 3d) in Ag foil exhibits peaks at 374.8 eV (3*d*_3/2_) and 368.8 eV(3*d*_5/2_), indicating a metallic Ag^0^ state. The Ag 3*d*_3/2_ and Ag 3*d*_5/2_ spectra of the Ag5ZnNS‐HF, the Ag10ZnNS‐HF, and the Ag30ZnMP‐HF samples are shifted slightly toward lower binding energy. This can be explained by the strong electron interaction between Ag and Zn, wherein Zn donates electrons Ag in the AgZnNS‐HF samples during the galvanic replacement process.^[^ ^30^ ^]^ The surface atomic ratio of AgZnNS‐HF samples was calculated via XPS and EDX, summarized in Table S1, Supporting Information. With the increase of GRR time, the surface atomic ratio of Ag increased to 29.2% (measured by XPS) in the Ag30ZnNS‐HF sample. The surface atomic ratio results calculated from XPS and EDX are similar, and the value for Ag increased with increasing galvanic replacement time.

The deconvoluted signals for XPS O 1*s* spectra of these samples consisted of three different peaks at 531.5, 533.1, and 535.1 eV (Figure 3e). The binding energy peak at 531.5 eV is associated with the M‐O species, while another peak at 533.1 eV, usually corresponds to the OH^−^ species. The binding energy peak at 535.1 eV can be attributed to the adsorbed water on the electrode surface. The moisture and the oxidation species detected via XPS analysis are attributed to the oxidized metallic zinc after exposure to the air during the ex situ XPS test.^[^ ^31^ ^]^ The relative percentage ratios of element valence states based on the deconvolution of peak areas in the ZnNS‐HF, Ag5ZnNS‐HF, Ag10ZnNS‐HF, and Ag30ZnNS‐HF samples were calculated in Table S2, Supporting Information. These results confirm that the zinc in HF samples and the silver in AgZnNS‐HF samples are mainly in the metallic state.

### 2.2 Electrocatalytic CO_2_RR Performance

**Figure 4:**
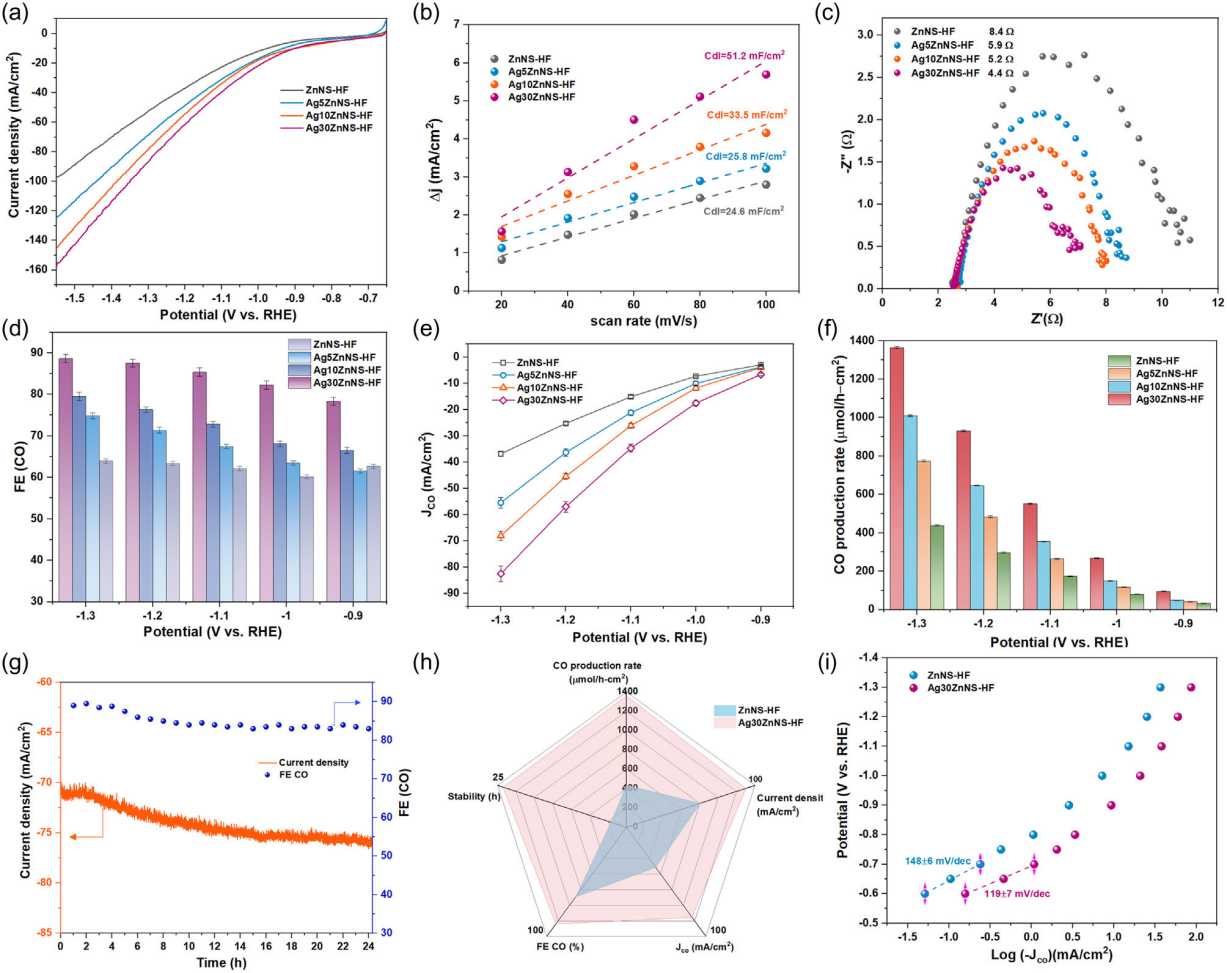
a) LSV of ZnNS‐HF, Ag5ZnNS‐HF, Ag10ZnNS‐HF, and Ag30ZnNS‐HF with CO_2_ purging through; b) dual‐layer capacitance (Cdl) of ZnNS‐HF, Ag5ZnNS‐HF, Ag10ZnNS‐HF, and Ag30ZnNS‐HF electrodes; c) Nyquist plots to evaluate electron resistance behavior of HFGDEs; d) FE of CO for ZnNS‐HF, Ag5ZnNS‐HF, Ag10ZnNS‐HF, and Ag30ZnNS‐HF electrodes; e) partial current density of CO for ZnNS‐HF, Ag5ZnNS‐HF, Ag10ZnNS‐HF, and Ag30ZnNS‐HF electrodes; f) production rate of CO for ZnNS‐HF, Ag5ZnNS‐HF, Ag10ZnNS‐HF, and Ag30ZnNS‐HF electrodes; g) long‐term operation of Ag30ZnNS‐HF electrode in flow‐cell for CO_2_RR in 0.5 M KCl; h) overall performance comparison for ZnNS‐HF and Ag30ZnNS‐HF; and i) Tafel plot of ZnNS‐HF and Ag30ZnNS‐HF.

The HFGDEs were tested for CO_2_RR in a customized H‐cell to determine their activity for electrochemical conversion of CO_2_. The electrocatalytic activity of HFGDEs was evaluated by liner scanning voltammetry (LSV) at a 5 mV s^−1^ scan rate, shown in **Figure** **4**a. The Ag30ZnNS‐HF exhibited a higher current density at a wide potential range from −0.7 to −1.55 V (versus reversible hydrogen electrode (RHE)), and it reached 157.8 mA cm^−2^ at −1.55 V (versus RHE). The high current density is attributed to higher contents of silver catalyst (29.2% measured by XPS) and the hierarchically silver sub‐nanosheets interconnected with the zinc nanosheets network, which provides increased ECSA and more active catalytic sites for CO_2_RR. The dual‐layer capacitance (Cdl) of the HFGDEs was calculated from the cyclic voltammetry cycles at different scan rates, presented in Figure 4b. The Cdl value increased with increasing the GRR time to improve the silver catalyst content on the outer surface of HFGDEs. The Ag30ZnNS‐HF electrode possessed the highest Cdl value (51.2 mF cm^−2^), which is more than twice the value of ZnNS‐HF. This confirms that the larger ECSA and the high content of silver catalysts can lead to a higher current density. In addition, the electrochemical impedance spectroscopy (EIS) test was conducted at −1 V (versus RHE) to evaluate the kinetics of charge transfer for HFGDEs (Figure 4c). The results showed a similar spectrum for HFGDEs but with different impedance arc sizes. The impedance arc sizes of AgZnNS‐HF electrodes became smaller compared to the ZnNS‐HF electrode, indicating the addition of silver catalyst could increase the electron transfer and reduce the charge transfer resistance. The impedance charge transfer value of the Ag30ZnNS‐HF was only 4.4 Ω, nearly half that of ZnNS‐HF, indicating the fast electron transfer in this electrode. The reduced charge transfer impedance is due to the hierarchically silver sub‐nanosheets interconnected with the zinc nanosheets network, providing multiple charge transfer channels to promote electron transfer and enhance the CO_2_RR kinetics.

To compare the CO_2_RR performance on the different HFGDEs, CO_2_RR tests were conducted at a potential range from −0.9 to −1.3 V (versus RHE) in a CO_2_‐saturated 0.5 M KCl electrolyte. Here, KCl was selected as the electrolyte because the adsorption of halide ions such as Cl^−^ could modulate the electrical states to suppress HER and facilitate the electrochemical conversion of CO_2_.^[^ ^32^ ^]^ The main products were H_2_, CO, and formate for the Cu HF substrate (Figure S9, Supporting Information). The FE for H_2_ was below 20% at −0.9 to −1.3 V (versus RHE), indicating a good CO_2_RR activity of bare Cu HF. However, the FE of CO and formate was around 40%, similar to that of copper hollow fiber electrodes with a relatively large Cu particle size.^[^ ^33^ ^]^ CO was the main product of AgZn catalyst‐modified HFGDEs, and the FE of CO increased with applied more negative potential (Figure 4d). Only a small amount of formic acid with a FE of less than 2% was detected by a high‐performance liquid chromatography (HPLC). The Ag30ZnMP‐HF electrode showed the highest FE of CO of 88.6% at −1.3 V (versus RHE). Compared to ZnNS‐HF with considerable Zn (002) facets that favor the HER, the growth of silver catalyst could suppress the HER, and the electronic interaction between Zn and Ag could increase the adsorption of the intermediates COOH* to promote the electrochemical conversion CO_2_ to CO. The partial current densities of CO (*J*_co_) for the HFGDEs (Figure 4e) showed a similar trend with current density and FE for CO. The *J*_co_ of the Ag30ZnNS‐HF reached 82.5 mA cm^−2^, which is more than twice the value of 36.8 mA cm^−2^ of the ZnNS‐HF. This is attributed to the hierarchically silver sub‐nanosheets interconnected zinc nanosheet network, which provides numerous catalytic sites, and the synergistic effect of Ag–Zn to promote the binding energy of COOH* intermediates.

Production rate is also an essential factor to consider in meeting the industry application requirement of CO production. The CO production rate for HFGDEs (Figure 4f) increased with the application of larger negative potentials, which aligns with the current density trend. The Ag30ZnNS‐HF can produce a 1364.6 μmol h^−1^ cm^−2^ CO at −1.3 V (versus RHE), 1009.2 μmol h^−1^ cm^−2^ for Ag10ZnNS‐HF electrode, 774.0 μmol h^−1^ cm^−2^ for Ag5ZnNS‐HF, and 438.9 μmol h^−1^ cm^−2^ for ZnNS‐HF electrode. We have also summarized the state‐of‐the‐art zinc‐based electrocatalyst with conventional planar electrodes used in H‐Cell or Zn‐based GDE used in flow‐type cells for the electrochemical reduction of CO_2_ to CO in Table S3, Supporting Information. The Zn‐based electrocatalyst exhibited a high FE of over 80% but suffered from low current density and CO production. The current density was below 50 mA cm^−2^, with most around 10 mA cm^−2^. The CO production rate of these catalysts was below 420 μmol h^−1^ cm^−2^ at a given potential. The relatively low CO_2_RR activity in H‐Cell is mainly attributed to the low local CO_2_ concentration and increased mass transportation resistance. The Ag30ZnHF exhibited much improved current density around 100 mA cm^−2^, a FE of over 85%, and a CO production rate over 1000 μmol h^−1^ cm^−2^ in H‐Cell. The improved CO_2_RR activity by applying HFGDE is due to the change of the gas delivery from nongas diffusion to gas diffusion. Unlike conventional GDEs requiring a separate gas chamber and incorporation of the flow cell, the CO_2_ gas can be fed directly into the lumen side of the hollow fiber and penetrated through the hollow fiber walls, reaching active sites, thus providing sufficient CO_2_ near the triple‐phase interfaces. The shorted diffusion path from the CO_2_ gas phase to the electrode surface is only about 50 nm, while the diffusion path in the non‐GDE conditions from bulk electrolytes to the electrode surface is about 50 μm.^[^ ^34^ ^]^ In the meanwhile, the Zn‐based GDE incorporated with flow cells can operate at a current density of over 200 mA cm^−2^ and achieve more than 84% FE for CO with a CO production rate over 3000 μmol h^−1^ cm^−2^. It should be noted that the studies with higher current density and CO production rate in aqueous media have employed alkaline electrolytes or higher concentrations of KHCO_3_, therefore the electron transfer resistance is substantially decreased and higher current densities can be achieved. This showcases the promise of using HFGDE to achieve high CO_2_RR performance operating at high current density and high concentrations of electrolytes.

The stability of the electrocatalyst is another crucial factor considered for commercial application. The long‐term stability of the Ag30ZnNS‐HF for CO_2_RR performance was conducted in a customized flow cell (Figure S10, Supporting Information). The CO_2_ flow rate was kept at 30 mL min^−1^, and the electrolytes (0.5 M KCl) were circulated at 10 mL min^−1^ in both anode and cathode chambers. The potential applied was −1.2 V (versus RHE) for the 24‐h stability of the Ag30ZnNS‐HF electrode (Figure 4g). In the initial four hours, the current density of the Ag30ZnNS‐HF was maintained at around 71 mA cm^−2^, and the FE for CO was around 88%. The current density increased to 75 mA cm^−2^, and the FE for CO dropped to 84% after 12 h. The current density was maintained at around 75 mA cm^−2^, and the FE for CO was maintained at around 83% in the last 12 h. Overall, the Ag30ZnNS‐HF exhibited a stable performance for current density and FE of CO (>83%) throughout 24 h at −1.2 V (versus RHE). In contrast, we also conducted the stability test for the ZnNS‐HF electrode (Figure S11a, Supporting Information). The current density increased from 39 to 41 mA cm^−2^, and the FE for CO dropped from 64% to 60% at the initial 3 h. Then, the current density increased to 47 mA cm^−2^, and the FE for CO dropped to 47% after 10 h, indicating a high HER of the ZnNS‐HF electrode. From the SEM image (Figure S11b, Supporting Information) of the electrode surface for the ZnNS‐HF electrode, the zinc nanosheet surface was covered with some bulk particles. The reason for the drop in CO_2_RR activity of ZnNS‐HF is possibly due to the carbonate salt precipitation or the formation of zinc hydroxide, which could block the pore structure and interrupt active catalytic sites. The Ag30ZnNS‐HF electrode retained the most silver sub‐nanosheets interconnected zinc nanosheets structure (Figure S12a, Supporting Information), and the XRD pattern (Figure S12b, Supporting Information) after the reaction also demonstrated the existence of Ag (111) crystal facet, the Zn (002), and Zn (100) crystal facets, similar to the XRD pattern before the reaction. In addition, the ZncNS‐HF electrode from our previous work,^[^ ^35^ ^]^ with only zinc nanosheet catalysts exhibited a limited 6 h stability at −1.1 V (versus RHE). The improved stability of Ag30ZnNS‐HF is attributed to the well‐connected silver sub‐nanosheets with zinc nanosheets, and the synergistic interaction between Ag and Zn to stabilize the Ag30ZnNS catalyst.

The overall performance comparison for the ZnNS‐HF and Ag30NS‐HF was presented in Figure 4h. The Ag30NS‐HF exhibited excellent CO_2_RR activity and stability, the value of production rate, and the partial current density of CO is more than twice that of ZnNS‐HF, and it also showed a promising 24‐h stability. For the electrochemical reduction of CO_2_ to CO, three reaction steps were widely acknowledged in the following Equation ((1), (2))–(3)

(1) $${\text{CO}}_{2}\left(\text{aq}\right)+\;\left[H^{+}+e^{-}\right]\to {\text{COOH}}^{*}\left(\text{aq}\right)$$

(2) $${\text{COOH}}^{*}\left(\text{aq}\right)+\left[H^{+}+\;e^{-}\right]\to {\text{CO}}^{*}\left(\text{aq}\right)+\;H_{2}O\;\left(\text{aq}\right)$$

(3) $${\text{CO}}^{*}\left(\text{aq}\right)\to \text{CO}\;\left(g\right)$$

CO_2_ is first adsorbed on the surface of the electrode and reduced to CO_2_* via one electron from the electrochemical process and then to the intermediate COOH* via a proton transfer step. Lastly, most of the COOH* intermediates are reduced with a proton and an electron to CO* that desorbs from metal catalysts to produce the main product CO.^[^ ^36^ ^]^ The Tafel slope was used to analyze the CO_2_ to CO conversion kinetics for HFGDEs to gain insights into the underlying mechanism of CO formation in CO_2_RR (Figure 4i). It is well known that the Tafel slope with a value of 118 mV dec^−1^ indicates the rate‐limiting step during CO_2_RR is the initial one electron transfer step (step (1)). The Tafel slope value of Ag30ZnNS‐HF was 119 mV dec^−1^, which is closer to the value of 118 mV dec^−1^ and much smaller than that of the ZnNS‐HF (148 mV dec^−1^). Therefore, the rate‐limiting step for these two electrodes is the initial one electron transfer step, and the Ag30ZnNS‐HF exhibited higher catalytic activity toward CO formation due to the faster electron transfer for CO_2_ molecule activation. This result is also aligned with the smaller interfacial charge transfer resistance observed for Ag30ZnNS‐HF, as compared to ZnNS‐HF and other AgZnNS‐HF electrodes (Figure 4c), showing improved electron transfer and kinetics of reaction on Ag30ZnNS electrode. The other reason for the high CO_2_RR activity for the Ag30ZnNS‐HF electrode is the improved stabilization of CO_2_ and the stronger adsorption of COOH* intermediate on the Ag30ZnNS‐HF electrode surface. Previous density functional theory (DFT) studies^[^ ^37^ ^]^ have revealed the COOH* binding energy as a critical descriptor for CO production, and Ag and Zn exhibit weak and strong COOH* binding energy, respectively. Therefore, the synergistic Ag30ZnNS can provide adequate COOH* binding energy for CO production, and the low charge transfer resistance could promote faster electron transfer to the adsorbed CO_2_ molecules on the electrode surface.

### 2.3 The Mechanism of CO_2_ Delivery in HFGDEs

**Figure 5:**
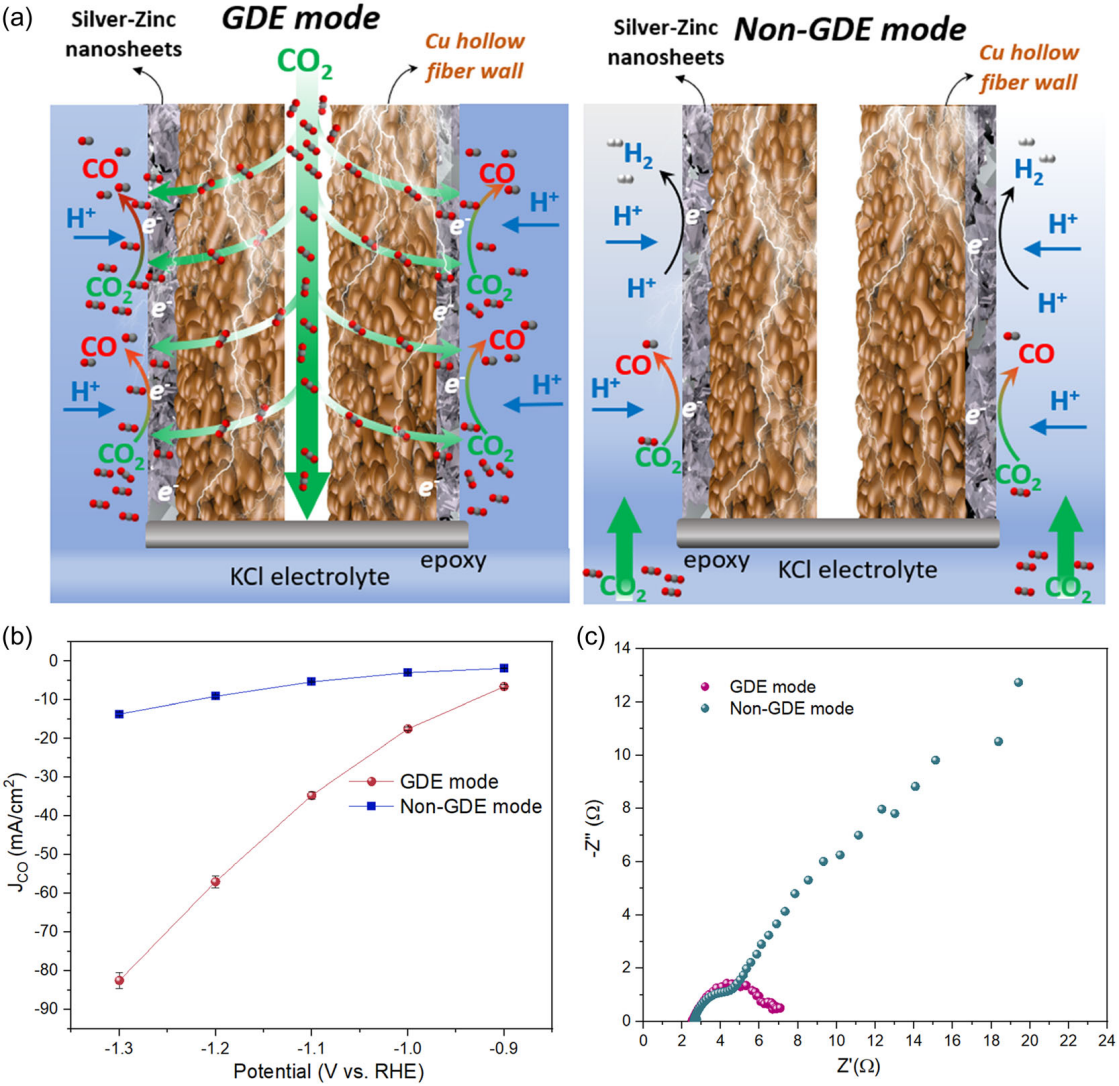
a) Schematic illustrations of CO_2_RR performance over Ag30ZnNS‐HF electrode in GDE mode and non‐GDE mode; b) CO partial current densities; and c) EIS Nyquist plots over Ag30ZnNS‐HF electrode in GDE mode and non‐GDE mode.

The gas disperses configuration of HFGDEs is flow‐through or gas penetration, meaning a pressure building up in the lumen side of the tube pushes the gas to penetrate through the hollow fiber wall to the active sites. This differs from the planar electrode, where gas dissolves in the electrolyte and diffuses to the catalyst/electrolyte interface, forming a gas concentration gradient from bulk electrolytes to the electrode surface. The CO_2_ concentration near the electrode is critical for CO_2_ reduction, particularly under high current densities, where the rapid energy supply causes significant CO_2_ consumption. The hierarchical porous structures in the HFGDE enable them to provide sufficient CO_2_ to the catalyst/electrolyte interface and promote the electrochemical reduction of CO_2_. To further investigate the CO_2_RR mechanism in the Ag30ZnNS‐HF electrode with different gas disperses configurations, we also tested the Ag30ZnNS‐HF electrode in non‐GDE mode, which directly put the CO_2_ gas outlet into the electrolyte, as shown in **Figure** **5**a, the CO_2_ is dissolved in the electrolyte. The partial current density of CO for the Ag30ZnNS‐HF electrode increased with more negative potential applied. The electrode in GDE mode showed a much higher CO partial current density of 82.5 mA cm^−2^ at −1.3 V (versus RHE), which is 6 times as high as the 13.6 mA cm^−2^ of the electrode in non‐GDE mode (Figure 5b). As revealed by the EIS measurement (Figure 5c), the Ag30ZnNS‐HF in GDE‐mode and non‐GDE mode showed a different charge transfer mechanism, as the kinetic control region (electrolyte resistance + charge transfer resistance) appeared in the GDE‐mode. In contrast, the electrode in non‐GDE mode showed a mass transfer control region (diffuse layer resistance). This caused a much higher charge transfer resistance for the electrode in non‐GDE mode, and the Ag30ZnNS‐HF electrode only has a charge transfer resistance of 4.4 Ω, confirming a fast electron transfer and improved kinetics in GDE mode.

## 3 Conclusion

In this study, the metallic microtubular GDEs were fabricated with nanoengineering a silver sub‐nanosheets supported on zinc nanosheets catalyst for electrochemical conversion of CO_2_ to CO. It was found that the in situ growth of silver sub‐nanosheets could provide more ECSA and increase the electron transfer channels to reduce the charge transfer resistance. The synergistic effect between Ag and Zn improved the adsorption binding energy of the COOH* intermediate, resulting in fast CO_2_RR kinetics. The porous metallic HFGDE can serve as a good electron conductor and gas diffuser, providing sufficient CO_2_ to catalyst/electrolyte interfaces and stabilizing the high CO_2_ local concentration near the electrode. The as‐prepared Ag30ZnNS‐HF exhibited a high partial current density of 82.5 mA cm^−2^ and a high CO production rate of 1364.5 μmol h^−1^ cm^−2^ at applied potential −1.3 V (versus RHE). Moreover, the Ag30ZnNS‐HF electrode was tested in both GDE and non‐GDE modes, and it showed a much higher partial current density of CO in GDE mode, which is more than 6 times higher than that of this electrode in non‐GDE mode. The charge transfer resistance in GDE mode was much smaller, as the electrode in non‐GDE mode exhibited a high diffusion layer resistance. The combination of a highly selective AgZn bimetal catalyst with a hierarchical nanosheet structure and a hollow fiber gas diffusion electrode configuration with a superior gas delivery property affords efficient CO_2_RR performance in producing CO. This study revealed the potential to design a stable, structured electrode for CO production by engineering a hollow fiber electrode and nanostructuring highly efficient electrocatalysts with hierarchical structure and reduced charge transfer resistance.

## Conflict of Interest

The authors declare no conflict of interest.

## Supporting information

Supplementary Material

## Data Availability

The data that support the findings of this study are available from the corresponding author upon reasonable request.
