# Supplementary material for: In Situ Growth of Hierarchical Silver Sub‐Nanosheets on Zinc Nanosheets‐Based Hollow Fiber Gas‐Diffusion Electrodes for Electrochemical CO2 Reduction to CO
Source: Small Sci. 2024 Jul 10;4(10):2400184. doi: 10.1002/smsc.202400184 (PMC11935171; doi:10.1002/smsc.202400184)
Supplement: Supplementary file 1 — Supplementary Material [file SMSC-4-2400184-s001.pdf]

Supporting Information

In situ growth hierarchical silver sub-nanosheets on zinc nanosheets based hollow fiber gas-diffusion electrodes for electrochemical CO_2 reduction to CO

Guoliang Chen, Lei Ge, Yizhu Kuang, Hesamoddin Rabiee*, Beibei Ma, Fatereh Dorosti, Ashok Kumar Nanjundan, Zhonghua Zhu and Hao Wang**

Experimental Section

Chemicals and Materials.

Cu powder (99% purity, 5μm) was purchased from Shanghai Xiangtian Nano Materials Co., Polyethersulfone (PES Ultrason E 6020 P) was purchased from BASF, Germany. Ag and Zn foil (99.9%, 0.1 mm) were purchased from Yudingda Metal Company, China. N-methyl-2-pyrrolidone (NMP), Zinc sulfate heptahydrate ($ZnSO_4·7H_2O$, 99% purity), Silver nitrate ($AgNO_3$, 99% purity), Potassium sulphate (K_2SO_4, 99.7% purity), Potassium chloride (KCl, 99% purity), phosphoric acid (H_3PO_4, 85% purity) were purchased from Sigma-Aldrich Australia. Nafion 117 membranes were purchased from Chemours company. Ultra-pure deionized water (18.2 MΩ·cm) used in all the experiments was obtained from the Milli-Q® Direct 8 system. Carbon dioxide (99.999% purity), Argon (99.999% purity), and 5%H_2/Ar were purchased from BOC gas company, Australia.

Fabrication of Cu hollow fiber (Cu HF).

The Cu HFs were fabricated via dry-wet spinning and sintering process, as shown in Fig. S1. Typically, to get a homogenous polymeric dope, the Cu powder (65wt%), PES (8.75wt%), and NMP (26.25wt%) were mixed in the ball mill jar for 24 h at 300 rpm. Then, the paste was vacuumed for 4h at room temperature to remove the bubbles before slowly pouring it into a stainless-steel vessel. To start the extrusion process, the paste was pushed through a spinneret rig, and the bore fluid (DI water) as inner coagulant liquid was pumped through the bore of the spinneret. In the meantime, the phase inversion outside of the hollow fiber starts once the fiber comes down to the water bath. To remove the NMP entirely, the fibers must be kept in the bath overnight. After that, the fibers were dried at room temperature for 24 h. To fully reduce Cu hollow fiber with good strength, the green fibers were first calcinated in the air at 600 °C for 3h

to remove PES. After that, the copper oxide fibers were taken out and immersed into a 0.2 M $NaBH_4$ solution for 3 h. Lastly, the fibers were calcinated in 5%H_2/Ar at 600 °C for 3h to fully reduce and increase their strength. The fibers were stored in the N_2 atmosphere before further tests.

Zinc catalysts electrodeposition on Cu HF.

The growth of zinc nanosheets on Cu hollow fiber was performed on a three-electrode system. Before the deposition, the counter electrode, a piece of zinc plate (2×6 cm^2, thickness 0.1mm), was immersed into 1 M HNO_3 solution for 30 minutes to remove the oxide surface and then rinsed with ethanol and deionized water before drying at N_2. The Cu HFs were electropolished at 85% phosphoric acid at 2 V for 120 s before electrodeposition. To prepare the zinc catalysts-based hollow fiber electrode, the Cu hollow fiber was first immersed into a 0.2 M $ZnSO_4$ solution as a working electrode, and an Ag/AgCl with 3 M NaCl solutions worked as a reference electrode. Then, applied -1.4 V (vs. Ag/AgCl) on potentiostat, on time 1 s, off time 3 s, set 250 cycles. A nitrogen flow rate was kept purging into the electrolytes during the electrodeposition process. The sample was synthesized via the above steps named zinc nanosheet hollow fiber (ZnNS-HF). Afterwards, the samples were raised with deionized water multiple times before drying with N_2.

In-situ growth silver sub-nanosheets on ZnNS-HF.

The in-situ growth siler sub-nanosheets on ZnNS-HF is via galvanic replacement reaction (GRR). The ZnNS-HF was immersed in 10 mM $AgNO_3$ + 2.5 mM K_2SO_4 solution for different periods. Afterwards, the samples were raised with deionized water multiple times before drying with N_2. The ZnNS-HF after GRR, named silver zinc nanosheet hollow fiber (AgZnNS-HF); the ZnNS-HF after 5 s GRR time named Ag5ZnNS-HF; 10 s of GRR time named Ag10ZnNS-HF; 30 s of GRR time named Ag30ZnNS-HF.

Characterizations.

The surface and cross-section morphology of HFGDEs were observed by scanning electron microscopy (SEM, JOEL-7100 F) coupled with an energy-dispersive X-ray analyser (EDX) for elemental mapping. The nanostructures of zinc nanosheets were inspected by Field Emission Transmission Electron Microscope (FETEM, Hitachi HF 5000, Japan). The crystalline structures of the bulk phase HFGDEs were analyzed by X-ray diffraction (XRD, Bruker advanced X-ray diffractometer, Cu Kα (λ=1.5405Å) radiation source, Japan). X-ray

photoelectron spectroscopy (XPS) was applied to analyze the surface valence state and surface compositions of the HFGDEs. XPS was conducted on a Kratos Axis ULTRA XPS with a monochromatic Al Kα radiation source (1486.6 eV) at 15 kV (10 mA) and a hemispherical electron energy analyzer (165 nm). CASA® software was used to analyze the XPS data, calibrated to the C 1s signal at 284.4 eV as the reference.

Electrochemical measurements.

The electrochemical CO_2 electroreduction measurements were performed in a home-built H-cell using HFGDEs as working electrodes. The working electrode was stuck into a ¼ copper tube via conductive silver epoxy, and the other ends as well joints were sealed and covered with non-conductive and gas-tight epoxy, with an exposed length of 2 cm. A Pt mesh (3×3 cm^2) was used as a counter electrode. An Ag/AgCl (3 M NaCl, BASi, USA) as a reference electrode was put near the working electrode, the distance between these two was around 1 cm. Nafion 117 Proton Exchange Membrane was used to separate the anode and cathode chambers. The potentials applied on the potentiostat (Autolab PGSTA302N with FRA32M) were converted to the reversible hydrogen electrode (RHE) scale via E (V vs. RHE) = E (V vs. Ag/AgCl) + 0.209 +0.0591 pH. The potential in this manuscript was used in RHE scale without RS competence. The pH value for 0.5 M KCl was measured by a pH meter (TPS, WP-Plus), and the value is around 3.8 in CO_2-saturated 0.5 M KCl solution. A 0.5 M KCl solution was used as both cathode and anode electrolyte. Before the test, the H-Cell was vacuumed and kept purging with 20 ml/min CO_2 for 30 minutes. The working electrode was reduced at -1 V for 10 min to remove the zinc oxide due to the spontaneous oxidization when the electrode was exposed to the air.

The dual-layer capacitance (Cdl) of ZncNS-HF HFs was conducted to evaluate the electrochemically active surface area (ECSA) of the electrode at different scan rates starting from 20 to 100 mV/s via:

$$C_{dl} = J \Big/ \frac{dV}{dt} \quad \text{(S1)}$$

Here, C_{dl} is the capacitance, the current density J is at the center of 0.1 V electrochemical window, and dV/dt is the cyclic voltammetry (CV) scan rate. The CV scans were carried out at 0.5 M CO_2-saturated KCl solution while purging CO_2 through the HFGDE samples.

Liner scanning voltammetry (LSV), with the sweeping rate of 5 mV/s, and from – 0.65V to − 1.55 V (vs. RHE) were obtained in CO_2-saturated 0.5 M KCl after 30 minutes of gas purging. The electrochemical impedance spectroscopy (EIS) test was conducted at a relatively lower

potential of -1 V (vs. RHE) to evaluate the kinetics of charge transfer hollow fiber electrodes. Chrono amperometry (CA) was performed at potentials between − 0.9 and − 1.3 V (vs. RHE) to obtain the Faradaic efficiency (FE) of at different potentials. Gas and liquid samplings were done after 1000 s of applying potential and reaching the steady-state condition.

Gaseous products were analysed with a Shimadzu 2014 GC, equipped with a ShinCarbon packed column (ST 80/100, 2 mm ID, 1/8 OD Silico, Restek). H_2 was detected by a thermal conductivity detector (TCD) and the rest of the gases were detected on a flame ionization detector (FID). The FE of gaseous products were determined via the following equation:

$$FE_i = \frac{e_i \times F \times P \times V \times x_i}{J \times R \times T} \times 100\% \qquad \text{(S2)}$$

where, e_i is the electron transfer required to produce one mole of a gaseous product (2 for H_2 and CO), x_i is the concentration of the products in the reactor gas outlet flow measured by GC, V is the exit gas volumetric flow rate, measured by a digital flow meter, P is the atmospheric pressure (101.3 kPa), and J is the current (A) obtained from the potentiostat.

Liquid products were measured by high-performance liquid chromatography (HPLC) (Shimadzu, Hi-Plex H, 7.7 × 300 mm, 8 µm column, SPD-20A/20AV UV-Vis detector). Only trace amounts ($FE_{formate}$<2%) of formate were detected by HPLC at high overpotential.

The stability assessment was evaluated in a customized flow cell, employing peristaltic pumps to circulate electrolytes (0.5 M KCl) in the anodic and cathodic parts. Anode and cathode compartments had a volume of 54 ml, and two extra reservoir containers with a volume of 250 ml were used for catholyte and anolyte circulation. The circulation rate was set at 10 ml/min, with CO_2 purging maintained at 30 ml/min. Samplings of both liquid and gas were performed once every hour. The reported data for each electrochemical test is the average of three repeated measurements.

Calculation of CO_2 permeability.

The gas permeability of the HFGDEs was calculated by measuring gas flux and pressure drop across the hollow fibers and using the following equation:

$$\mathrm{P} = \frac{\mathrm{F.L}}{\mathrm{A\Delta p}} \qquad \text{(S3)}$$

where, P is the gas permeability [mol.m.m^{-2}.pa^{-1}.s^{-1}], F is molar flow rate [mol.s^{-1}], L is the HFGDE's wall thickness [µm], A is the HFGDE's outer surface area [m^2], and Δp is the

pressure drop [pa] across the hollow fiber (Δp is read from a pressure gauge installed on CO_2 line before the hollow fiber). P is reported in Barrer (1 Barrer = $3.35 \times 10^{-16} \frac{\text{mol.m}}{\text{m}^2\text{.pa.s}}$).

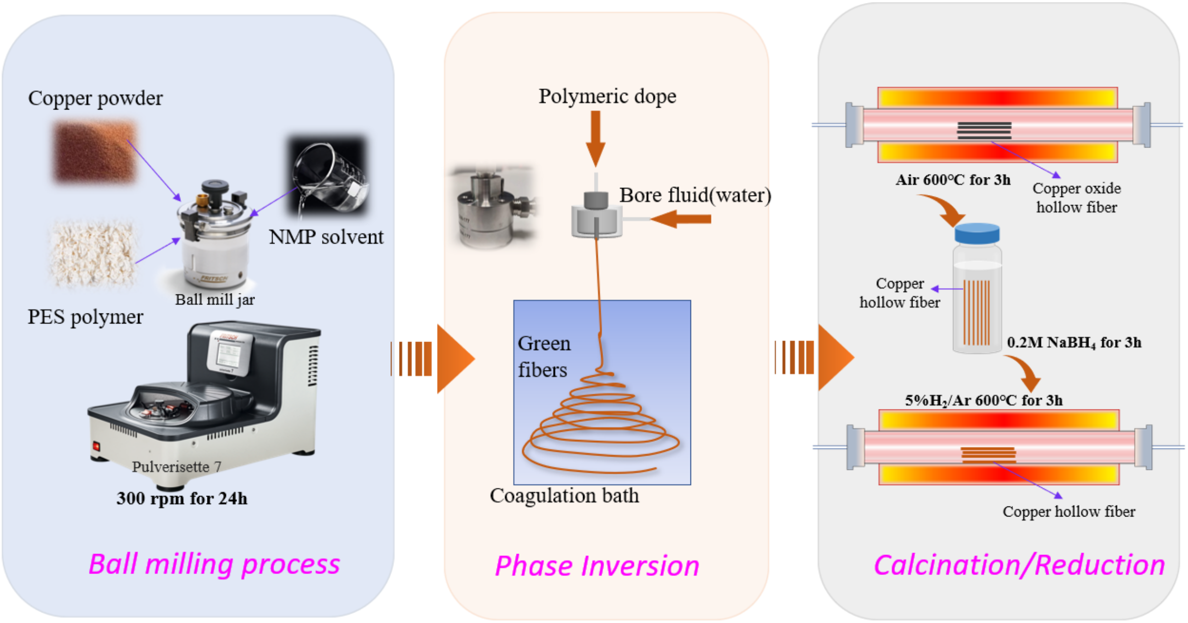

Figure S1. Schematic of copper hollow fiber fabrication process.

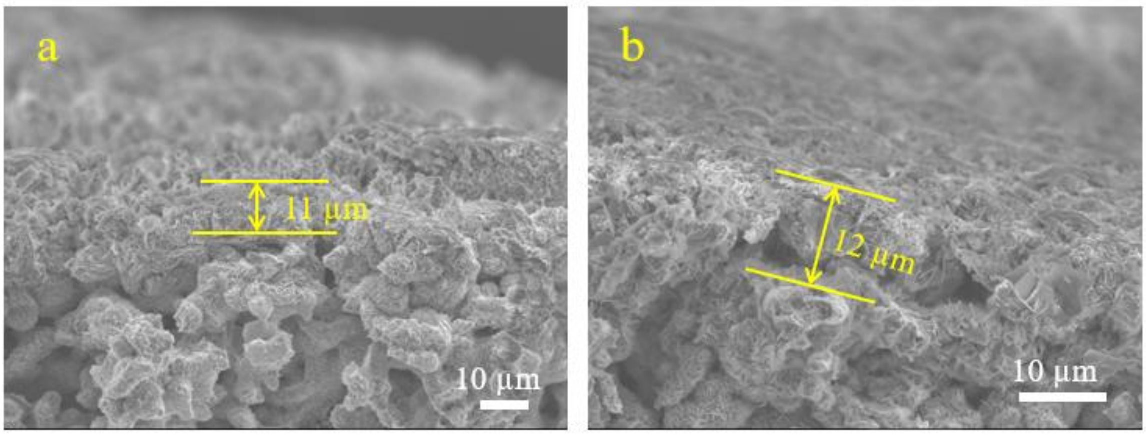

Figure S2. Cross-section SEM images of a) ZnNS-HF, and b) Ag30ZnNS-HF

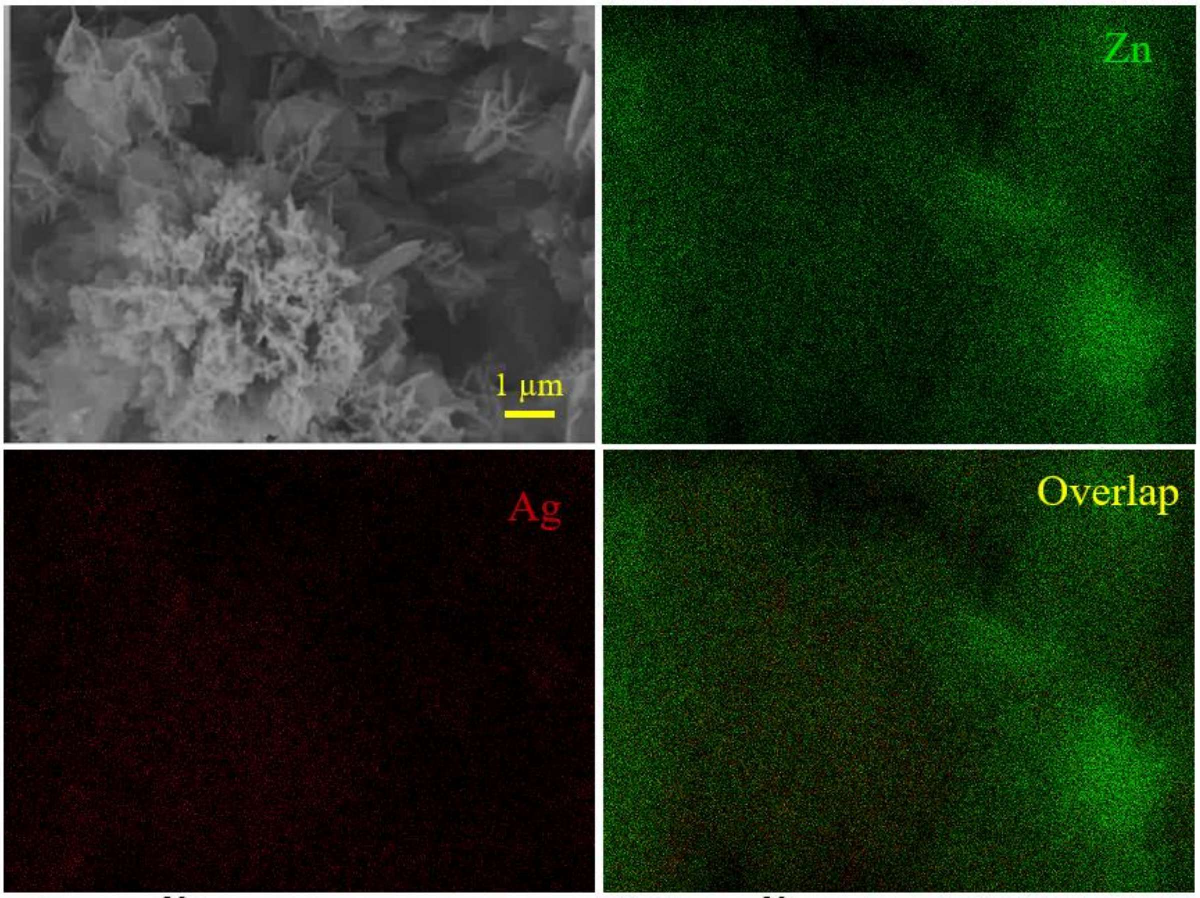

Figure S3 Element mapping images of Ag30ZnNS-HF

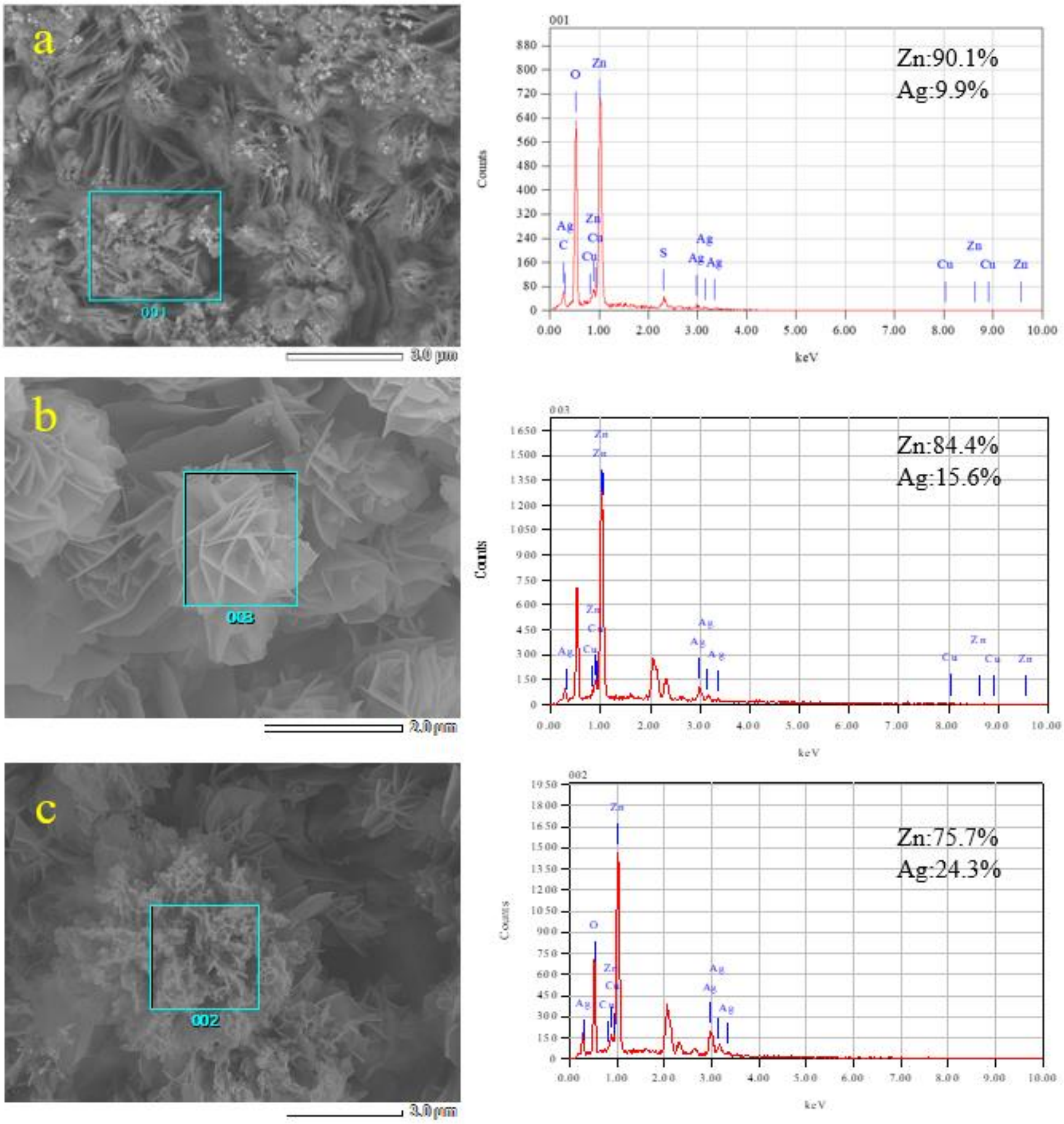

Figure S4. FESEM-EDS results of a) Ag5ZnNS-HF, b) Ag10ZnNS-HF, and d) Ag30ZnNS-HF

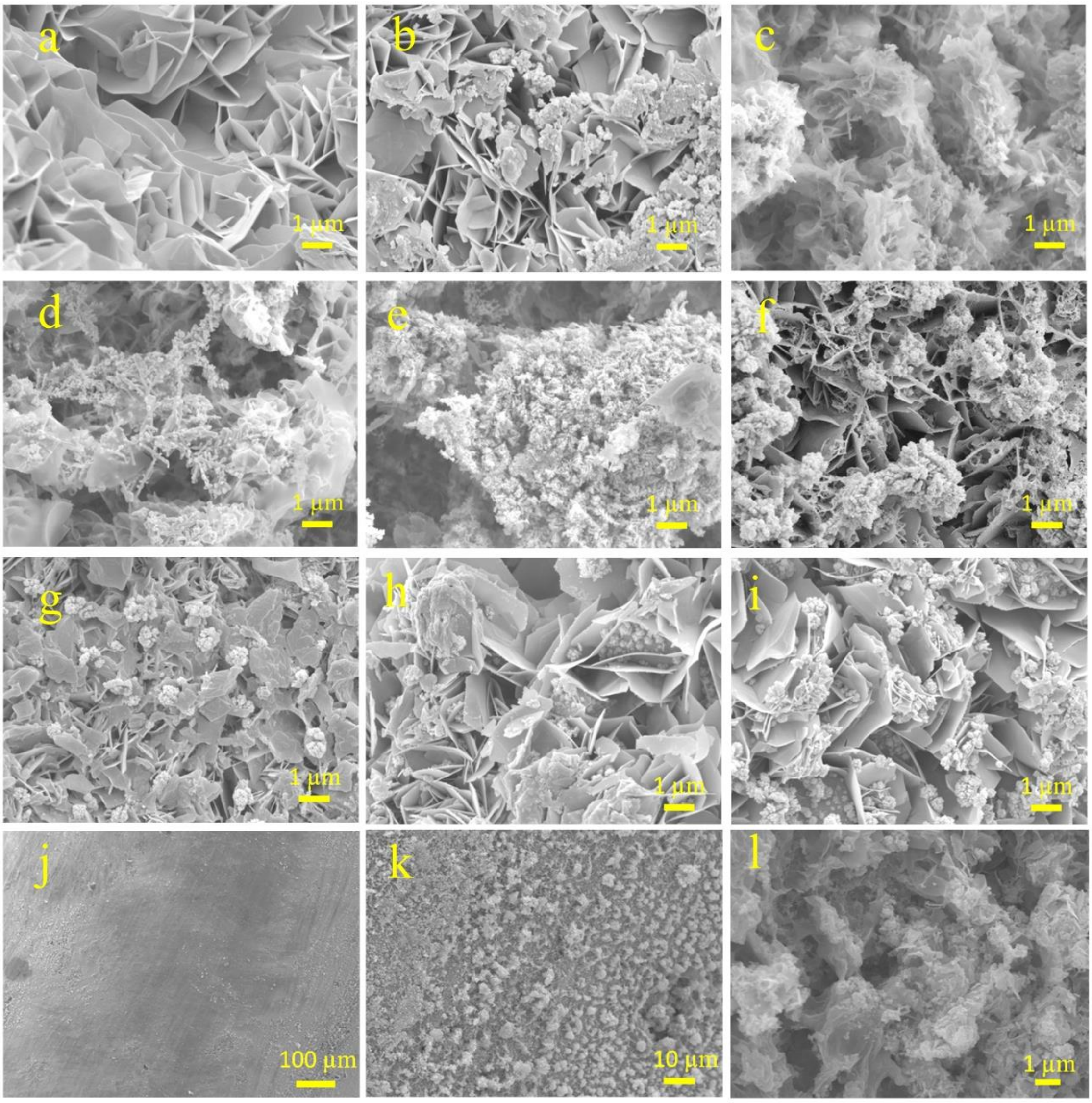

Figure S5. Surface SEM images of AgZnNS-HF sample at different preparation conditions, a) ZnNS-HF, galvanic replacing time of b)1 s, (c, d) 60 s, and e) 5 mins in 10 mM $AgNO_3$ + 5mM K_2SO_4 solution; f) galvanic replacing time of 30 s in 10 mM $AgNO_3$ + 2.5 mM K_2SO_4 solution; galvanic replacing time of 30 s in g) 1 mM $AgNO_3$, h) 3 mM $AgNO_3$, and i) 5 mM $AgNO_3$ solution; (j, k, and l) galvanic replacing time of 30 s in 10 mM $AgNO_3$ + 5mM K_2SO_4 solution with a flat Zn HF (Note: the flat Zn HF was made by carefully scratching the surface of ZnNS HF with a sharp blade)

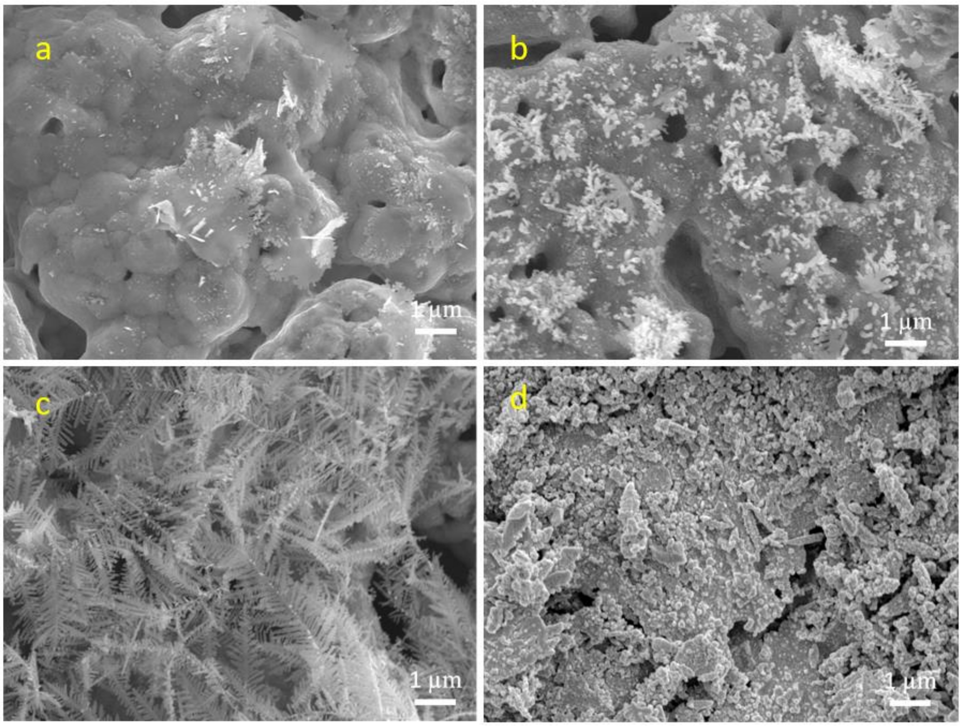

Figure S6. Surface SEM images of Cu HF samples at different GRR time and silver solutions, a)5 s, b) 10s, and c) 30 s in 10 mM $AgNO_3$ + 5mM K_2SO_4 solution; d) 30 s in 10 mM $AgNO_3$ solution

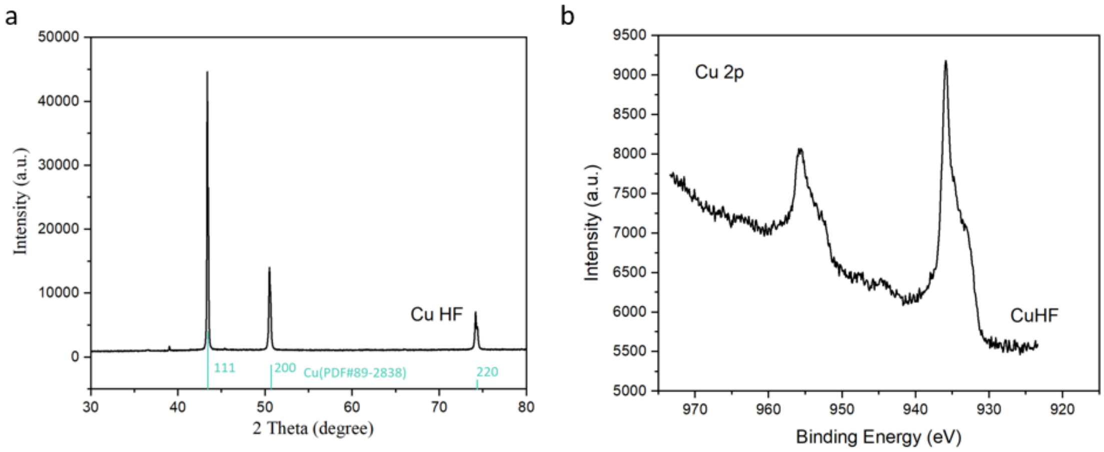

Figure S7. a) XRD pattern, and b) Cu 2p XPS spectrum for the origin Cu HF

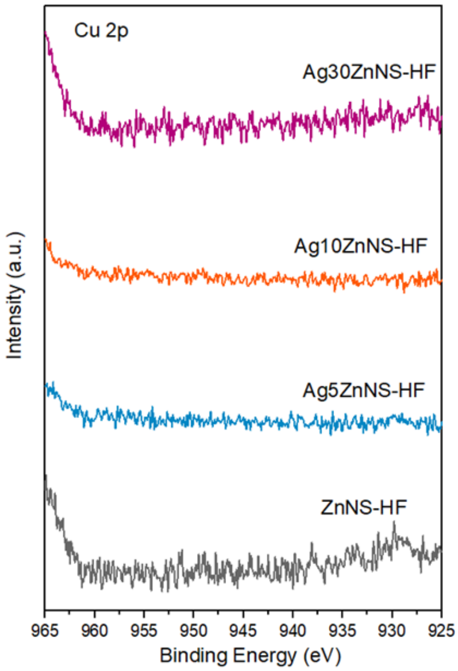

Figure S8. Cu 2p XPS spectrum for ZnNS-HF, Ag5ZnNS-HF, Ag10ZnNS-HF and Ag30ZnNS-HF samples.

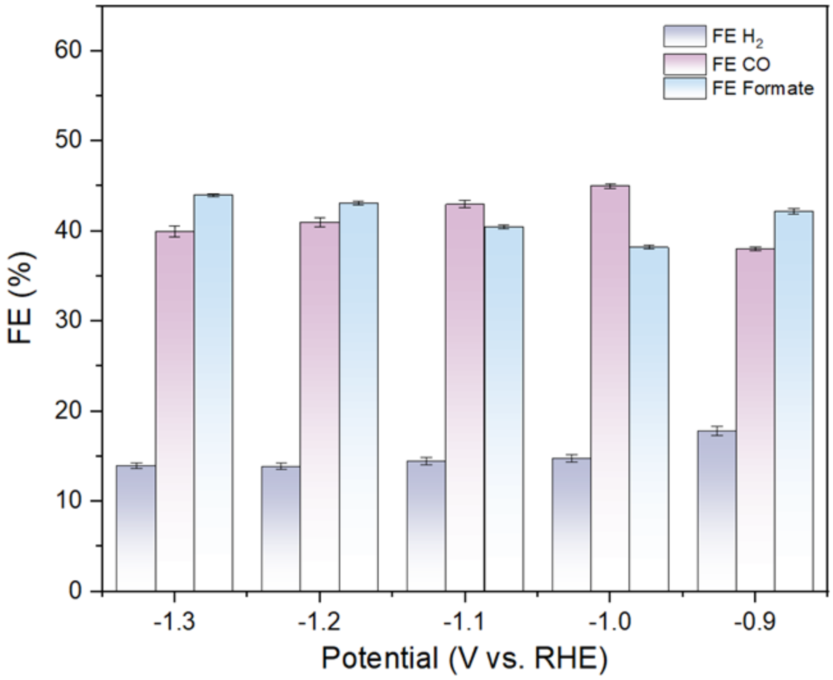

Figure S9. CO_2RR performance of bare Cu HF

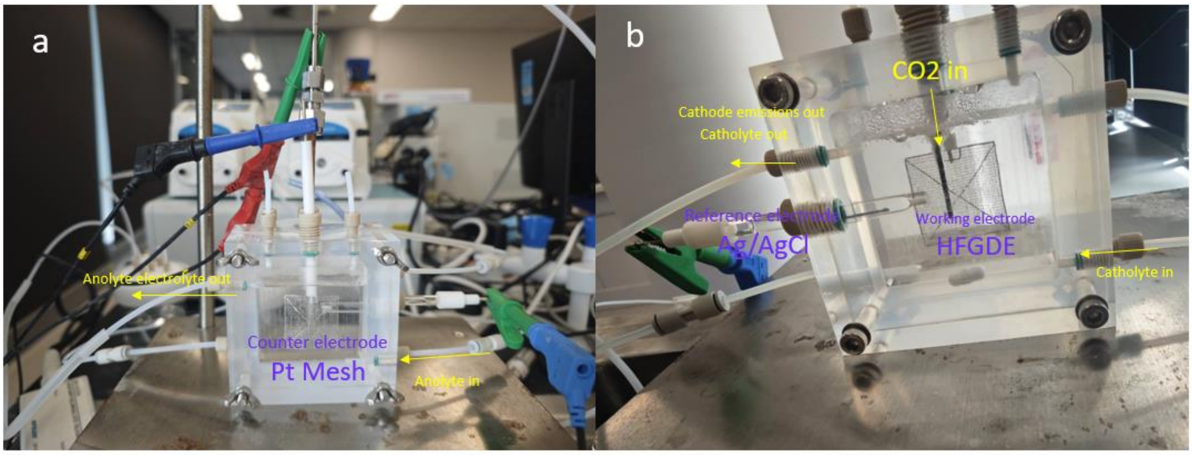

Figure S10. a) Optical images of a customized-flow cell (view in front of anode), and b) view in front of cathode

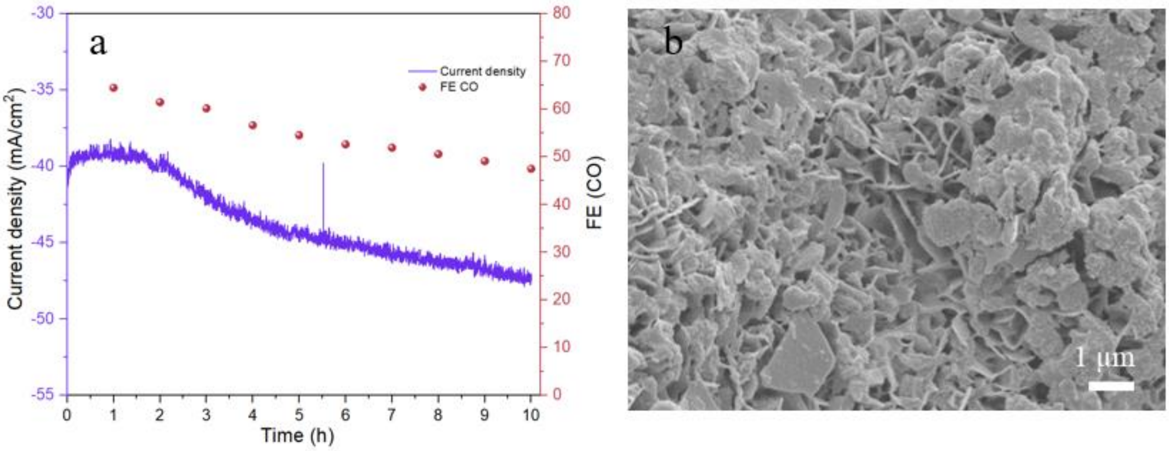

Figure S11. a) Long-stability test result (current density and FE CO) of ZnNS-HF, and b) surface SEM image of ZnNS-HF after stability test

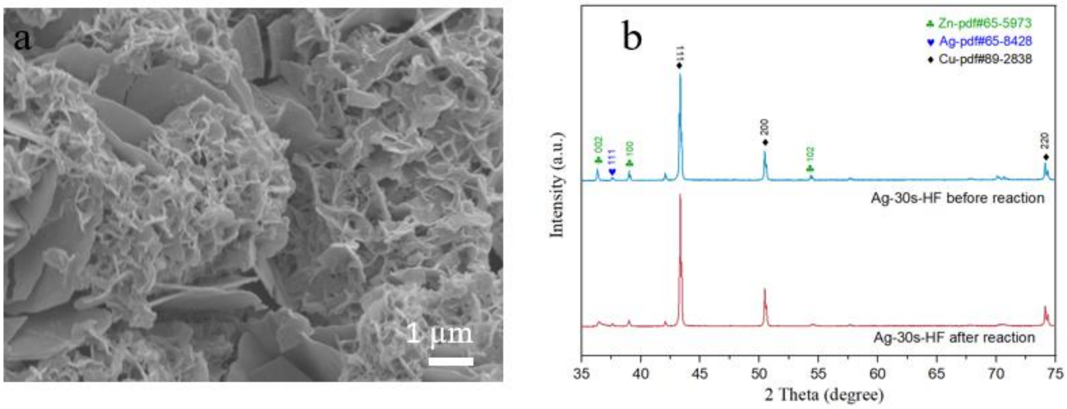

Figure S12. a) Surface SEM image of Ag30ZnNS-HF after stability test, and b) XRD pattern of Ag30ZnNS-HF before and after stability test

Table S1. Surface atomic ratio of Zn and Ag for AgZnNS-HF samples examined by EDS and XPS.

Samples	EDS		XPS	
	Zn (%)	Ag (%)	Zn (%)	Ag (%)
ZnNS-HF	100	0	100	0
Ag5ZnNS-HF	90.1	9.9	91.1	8.9
Ag10ZnNS-HF	84.4	15.6	86.0	14.0
Ag30ZnNS-HF	75.7	24.3	70.8	29.2

Table S2. The relative percentage ratio of element valence states based on the deconvolution of peak areas in the ZnNS-HF, Ag5ZnNS-HF, Ag10ZnNS-HF, and Ag30ZnNS-HF samples.

Samples	The peak area ratio of metal species with different valences (%)			
	(Zn $2p_{3/2}$+Zn $2p_{1/2}$) Zn^0/ (Zn $2p_{3/2}$+Zn $2p_{1/2}$) Zn^{2+}	Zn^0/Zn^{2+}	Adsorption/OH^- /M-O	Ag
ZnNS-HF	(56.8+29.7)/(7.7+5.8)	85.6/13.4	6.6/13.9/79.5	0
Ag5ZnNS-HF	(52.3+30.7)/(8.8+8.2)	83.0/17.0	9.2/23.6/67.2	100
Ag10ZnNS-HF	(45.3+26.7)/(16.6+11.4)	72.0/28.0	14.6/17.9/67.5	100
Ag30ZnNS-HF	(56.2+29.6)/(7.7+6.5)	85.8/14.2	5.6/18.8/75.6	100

Table S3. Performances of zinc-related electrocatalysts for CO_2RR to produce CO.

Material	Electrode geometry	Electrolyte	Operating Potential (V vs. RHE)	Current Density /mA/cm^2	FE CO	CO production rate/ μmol/h-cm^2	Ref
Ag30ZnNS-HF	Cu HF	0.5 M KCl	-1.3	93.1	88.6	1364.56	This work
	Cu HF	0.5 M KCl	-1.2	65.1	87.6	930.8	
Ag10ZnNS-HF	Cu HF	0.5 M KCl	-1.3	85.6	79.5	1009.2	
Ag5ZnNS-HF	Cu HF	0.5 M KCl	-1.3	74.0	74.9	774.0	
ZnNS-HF	Cu HF	0.5 M KCl	-1.3	57.6	63.9	438.9	
h-Zn	Zn foil	0.5 M $KHCO_3$	-1.1	24.4	83.6	379.9	[1]
Zn dendrite	Zn foil	0.5 M $NaHCO_3$	-1.1	17.0 a)	79	250.6 c)	[2]
Nanoscale Zn	Zn foil	0.5 M NaCl	-1.1	15.0 a)	93.0	260.3 c)	[3]
Zn electrode	Zn foil	0.1 M $KHCO_3$	-1.3	4.1	78.9	60.4 c)	[4]
H-Zn-NPs	Zn foil	0.1 M $KHCO_3$	-1.26	14.2 b)	91	240.6 c)	[5]
Porous Zn	Cu mesh	0.1 M $KHCO_3$	-0.95	13.4 b)	95	236.9 c)	[6]
$Zn_{94}Cu_6$ foam	Cu foil	0.5 M $KHCO_3$	-0.95	8.8 b)	90	147.4 c)	[7]
Zn nanosheets	Zn foil	0.5 M $KHCO_3$	-1.13	6.0 b)	86	97.0 c)	[8]
P-NS	Carbon paper	0.1 M $KHCO_3$	-1.2	15.6 b)	90 a)	261.2 c)	[9]
Zn-3	Cu foam	0.1 M $KHCO_3$	-1.3	42 a)	36 a)	282 a)	[10]
LD-ZnO	Zn foil	0.1 M $KHCO_3$	-1.6	34 b)	64 a)	406.1	[11]
Zn-1	Zn foil	0.1 M $KHCO_3$	-0.85	9.6	93.6	170	[12]
R-Zn-CO_2	Zn foil	0.5 M $NaHCO_3$	-0.9	6.6	77.8	95	[13]
V_0-rich ZnO	Carbon paper	0.1 M $KHCO_3$	-1.1	19	83	290	[14]
Zn-Ag 2	Glass carbon	0.1 M $KHCO_3$	-0.8	2.3 b)	84	29.8	[15]
Ag-NDs/Zn-NLs	Zn foil	0.1 M $KHCO_3$	-1.0	9.51 b)	85.2	151.1 c)	[16]
AZ/C/Ag	Zn foil	0.5 M $KHCO_3$	-1.0	8.5 b)	86	136.2	[17]
0.08Cu/ZnO	Carbon paper	0.1 M $KHCO_3$	-1.3	10 b)	94	175.3	[18]
ZnO-Ag@UC	Carbon paper	0.5 M $KHCO_3$	-0.93	22.7 b)	98.1	416.0	[19]
Cu_{25}Zn-A	Zn plate	0.5 M $KHCO_3$	-1.18	48.3 b)	79.9	720	[20]
P-Zn GDE d)	Cu Mesh	1 M KOH	-0.64	200	84	3133.9 c)	[6]
OD-$Zn_{2.5}$ d)	GDL	2 M $KHCO_3$	-0.95	200	84.8	3189.8 c)	[21]
OD-ZnO d)	Carbon paper	1 M KOH	-0.62	200	91.6	3400 c)	[12]
C/Ag/PTFE d)	PTFE	1 M KOH	-0.7	200	90	3385.4 c)	[22]
Ag/GDL d)	GDL	1 M KOH	-0.82	300	90	5078.1 c)	[23]
Ag/GDL d)	GDL	3 M KOH	-1.1	440	90	7447.9 c)	[24]

a) This value is not mentioned in the article but derived from the graphical results;

b) This value is not mentioned in the article but calculated from the partial current density of CO and Faradaic efficiency;

c) This value is not mentioned in the article but is calculated from the current density and Faradaic efficiency.

d) This sample was performed in flow-type cell.

Supplementary References

[1] D.H. Won, H. Shin, J. Koh, J. Chung, H.S. Lee, H. Kim, S.I. Woo, *Angew. Chem. Int. Ed. Engl.* **2016**, 128, 9443-9446.

[2] J. Rosen, G.S. Hutchings, Q. Lu, R.V. Forest, A. Morre, F. Jiao, *Acs Catal*. **2015**, 5, 4586-4591.

[3] F. Quan, D. Zhong, H. Song, F. Jia, L. Zhang, *J. Mater. Chem. A* **2015**, 3, 16409-16413.

[4] KP Kuhl, T Hatsukade, ER Cave, DN Abram, J Kibsgaard, TF Jaramillo, *J. Am. Chem. Soc*. **2014**, 136, 14107-14113.

[5] J. Xiao, M.R. Gao, S. Liu, J.L. Luo, *ACS Appl. Mater. Interfaces* **2020**, 12, 31431-31438.

[6] W. Luo, J. Zhang, M. Li, A. Züttel, *ACS Catal*. **2019**, 9, 3783-3791.

[7] P. Moreno-García, N. Schlegel, A. Zanetti, A. Cedeño López, M.J. Gálvez-Vázquez, A. Dutta, M. Rahaman, P. Broekmann, *ACS Appl. Mater. Interfaces* **2018**, 10, 31355-31365.

[8] T. Zhang, X. Li, Y. Qiu, P. Su, W. Xu, H. Zhong, H. Zhang, *J. Catal*. **2018**, 357,154-162.

[9] K. Liu, J. Wang, M. Shi, J. Yan, Q. Jiang, *Adv. Energy Mater.* **2019**, 9, 1900276.

[10] B. Qin, Y. Li, H. Fu, H. Wang, S. Chen, Z. Liu, F. Peng, *ACS Appl. Mater. Interfaces* **2018**, 10, 20530-20539.

[11] B. Qin, Q. Zhang, Y.H. Li, G. Yang, F. Peng, *ACS Appl. Mater. Interfaces* **2020**, 12, 30466-30473.

[12] W. Luo, Q. Zhang, J. Zhang, E. Moioli, K. Zhao, A. Züttel, *Appl. Catal. B* **2020**, 273, 119060.

[13] D.L.T. Nguyen, M.S. Jee, D.H. Won, H. Jung, H.S. Oh, B.K. Min, Y.J. Hwang, *ACS Sustainable Che. Eng.* **2017**, 5, 11377-11386.

[14] Z. Geng, X. Kong, W. Chen, H. Su, Y. Liu, F Cai, G. Wang, J. Zeng, *Angew. Chem. Int. Ed. Engl.* **2018**, 130, 6162-6167.

[15] Q Yu, X Meng, L Shi, H Liu, J Ye, *Chem. Commun*. **2016**, 52, 14105-14108.

[16] S. Yang, M. Jiang, M. Wang, L. Wang, X. Song, Y. Wang, Z. Tie, Z. Jin, *Nano Res.* **2023**, 16, 8910-8918.

[17] Y. Gao, F. Li, P. Zhou, Z. Wang, Z. Zheng, P. Wang, Y. Liu, Y. Dai, M.-H. Whangbo, B. Huang, *J. Mater. Chem. A* **2019**, 7,16685-16689.

[18] L. Xue, C. Zhang, T. Shi, S. Liu, H. Zhang, M. Sun, F. Liu, Y. Liu, Y. Wang, X. Gu, S. Zeng, *Chem. Eng. J.* **2023**, 452, 139701.

[19] Z. Zhang, G. Wen, D. Luo, B. Ren, Y. Zhu, R. Gao, H. Dou, G. Sun, M. Feng, Z. Bai, A. Yu, Z. Chen, *J. Am. Chem. Soc*. **2021**, 143, 6855-6864.

[20] J. Feng, J. Li, L. Qiao, D. Liu, P. Zhou, J. Ni, H. Pan, *Appl. Catal. B* **2023**, 330,122665.

[21] M.P.L. Kang, M.J. Kolb, F. Calle‐Vallejo, B.S. Yeo, *Adv. Funct. Mater*. **2022**, 32, 2111597.

[22] C.T. Dinh, F.P. García de. Arquer, D. Sinton, E.H. Sargent, *ACS Energy Lett*. **2018**, 3, 2835-2840.

[23] C.M. Gabardo, A. Seifitokaldani, J.P. Edwards, C.T. Dinh, T. Burdyny, M.G. Kibria, C.P. O'Brien, E.H. Sargent, D. Sinton, *Energy Environ. Sci.* **2018**, 11, 2531-2539.

[24] S. Verma, X. Lu, S. Ma, R.I. Masel, P.J.A Kenis, *Phys. Chem. Chem. Phys.* **2016**, 18, 7075–7084.
